# Pan‐cancer multi‐omics reveals DCAF7 as an immune‐modulating prognostic driver and Wnt/β‐catenin activator in hepatocellular carcinoma

**DOI:** 10.1002/ctm2.70572

**Published:** 2025-12-31

**Authors:** Ruina Luan, Hanbin Lin, Xin Zhao, Jianpeng Li, Maohe Chen, Shiping Luo, Xinjian Lin

**Affiliations:** ^1^ School of Basic Medical Sciences, Key Laboratory of Translational Tumor Medicine in Fujian Province Putian University Putian Fujian China; ^2^ Central Laboratory Affiliated Hospital of Putian University Putian Fujian China; ^3^ Department of Pediatric Orthopaedics Shengjing Hospital of China Medical University Shenyang Liaoning China; ^4^ Department of Breast Surgery Affiliated Hospital of Putian University Putian Fujian China; ^5^ Department of Breast Surgical Oncology Clinical Oncology School of Fujian Medical University, Fujian Cancer Hospital Fuzhou Fujian China; ^6^ Key Laboratory of Gastrointestinal Cancer (Fujian Medical University) Ministry of Education Fuzhou Fujian China

**Keywords:** DCAF7, hepatocellular carcinoma, immune infiltration, pan‐cancer, Wnt signalling

## Abstract

**Background:**

DDB1 and CUL4‐associated factor 7 (DCAF7) is a WD‐repeat adaptor that recruits substrates to the CUL4DDB1 ubiquitinligase complex, but its pan‐cancer relevance and mechanistic contribution to tumor progression remain unclear.

**Methods:**

Multi‐omics datasets (genomic, transcriptomic, epigenomic, proteomic and single‐cell) from 33 tumor types were integrated to define DCAF7 expression, regulation, and clinical significance. Somatic alterations and copy‐number variation were analysed across cohorts, and promoter methylation and RNA modification signatures were interrogated. Immune associations were assessed by computational deconvolution and checkpoint‐gene profiling. Pathway and network analyses were performed to infer DCAF7‐linked programmes. Mechanistic and functional validation was conducted in hepatocellular carcinoma (LIHC) cell lines (HepG2, Huh7) using DCAF7 perturbation and pharmacologic Wnt inhibition.

**Results:**

DCAF7 was overexpressed in most cancers, consistent with copy‐number gain, focal promoter hypomethylation and putative m^6^A‐linked post‐transcriptional regulation, whereas hypermethylation at two CpG loci predicted poor prognosis in LIHC. DCAF7 alterations, predominantly amplifications, were associated with shorter overall survival in LIHC and positively correlated with DCAF7 mRNA abundance across cohorts. Immunogenomic analyses linked high DCAF7 to CD4^+^ T‐cell enrichment, broad upregulation of checkpoint genes (PD‐1/PD‐L1, CTLA‐4, TIGIT), and increased tumour mutational burden, microsatellite instability and neoantigen load, suggesting an immune‐evasive phenotype. Network and enrichment analyses converged on canonical Wnt/β‐catenin, Hippo and cell‐cycle programs. In vitro, DCAF7 promoted LIHC cell proliferation and migration by stabilising β‐catenin via increased inhibitory Ser9 phosphorylation of GSK‐3β, thereby inducing c‐Myc and cyclin D1; DCAF7 knockdown or the Wnt inhibitor XAV939 attenuated these effects. Drug‐response modelling further predicted increased sensitivity of DCAF7‐high tumours to 17‐AAG, docetaxel and alsterpaullone.

**Conclusions:**

DCAF7 is frequently activated by genetic and epigenetic mechanisms across cancers, associates with an immunotherapy‐relevant tumour immune milieu, and drives Wnt/β‐catenindependent malignant phenotypes in LIHC. These findings support DCAF7 as a prognostic biomarker and a candidate therapeutic target, particularly for stratified intervention in LIHC.

**Key points:**

1. DCAF7 is up‐regulated in various tumours and correlates with poor prognosis, particularly in LIHC.
2. High DCAF7 expression is linked to CD4^+^ T cell infiltration, up‐regulation of immune checkpoint genes and increased tumour mutational burden, suggesting a role in tumour immune escape.
3. DCAF7 stabilises β‐catenin by enhancing GSK‐3β Ser9 phosphorylation, thereby driving c‐Myc/cyclin D1 expression and contributing to proliferation and migration in LIHC.
4. DCAF7‐high tumours demonstrate therapeutic vulnerability to 17‐AAG, docetaxel and CDK/GSK‐3 inhibitor, revealing potential targeted treatment strategies.

## Graphical abstract

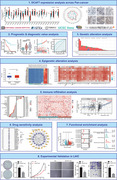

## 1 INTRODUCTION

Cancer remains a growing global health crisis, threatening both longevity and quality of life while imposing substantial economic burdens on patients and their families.^1^ By mid‐century, malignancies are projected to surpass cardiovascular diseases as the leading cause of premature mortality in most nations.^2^ Deciphering the molecular events that sustain cancer progression is therefore imperative.^3^ Pan‐cancer analyses, integrating multi‐omics data across tumour types, have emerged as powerful tools to uncover shared and lineage‐specific alterations, guiding the rational design of combination and precision therapies.^4^, ^5^, ^6^

Scaffold proteins, which organise higher‐order signalling complexes, are pivotal to cellular processes such as proliferation, survival, motility, immunity and differentiation.^7^, ^8^, ^9^ DDB1 and CUL4‐associated factor 7 (DCAF7), also known as WDR68 or HAN11, is a WD40‐repeat scaffold whose β‐propeller architecture presents multiple binding surfaces.^10^ Acting as a receptor for the CUL4–DDB1 E3 ubiquitin ligase, DCAF7 directs substrate ubiquitination to regulate protein stability.^11^ In pancreatic neuroendocrine tumours, the CUL4B–DCAF7 complex destabilises the tumour suppressor MEN1, thereby influencing cell growth and sensitivity to mTOR inhibition.^12^ DCAF7 can also scaffold deubiquitinase–substrate interactions. For instance, recruiting USP10 to G3BP1 in nasopharyngeal carcinoma, where de‐ubiquitination promotes chemoresistance and metastasis.^13^ Beyond the proteasome system, DCAF7 tethered to kinases DYRK1A and HIPK2 enhances hyperphosphorylation of adenovirus E1A, suppressing oncogenic transformation in HeLa cells.^14^

Despite these isolated observations, a systematic, pan‐cancer evaluation of DCAF7 has been lacking. Leveraging patient datasets from The Cancer Genome Atlas (TCGA), Genotype‐Tissue Expression (GTEx), Clinical Proteomic Tumor Analysis Consortium (CPTAC) and complementary resources, we examine DCAF7 expression, prognostic value, genetic alterations, epigenetic regulation, single‐cell function, immune‐cell infiltration and drug‐sensitivity profiles across 33 cancers. Focusing on liver hepatocellular carcinoma (LIHC), we identify DCAF7‐dependent transcriptional programs enriched for Wnt signalling, and we experimentally validate that DCAF7 drives LIHC cell growth and migration via Wnt/β‐catenin activation. Together, these results position DCAF7 as a central regulator of oncogenic signalling and tumour immunity with translational potential.

## 2 RESULTS

### 2.1 Pan‐cancer expression landscape and baseline distribution of DCAF7

**FIGURE 1:**
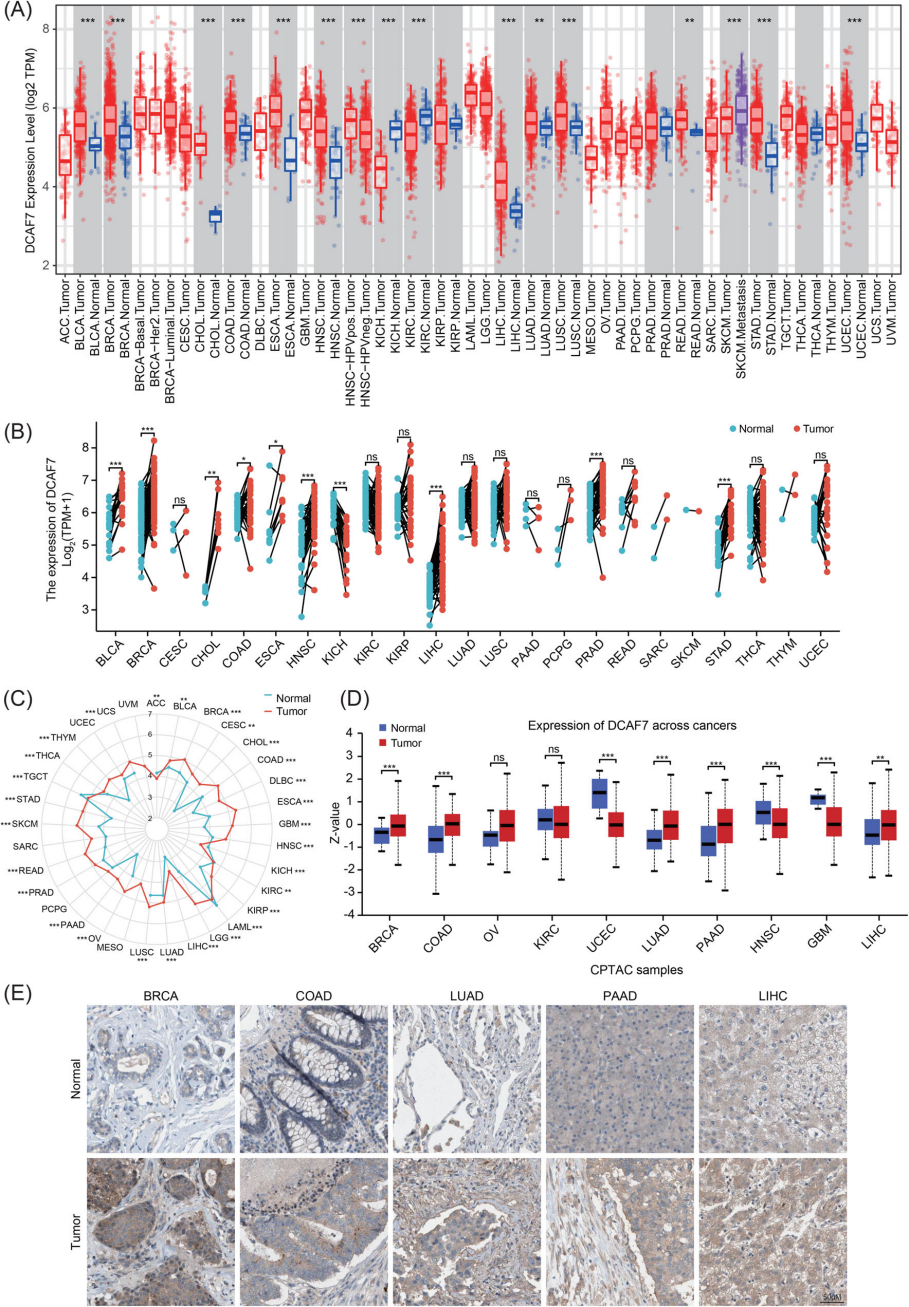
Pan‐cancer expression profile of DCAF7. (A) Differential mRNA abundance of DCAF7 in primary tumours versus normal tissues across TCGA cohorts (TIMER2.0). (B) Matched‐pair comparison of DCAF7 transcript levels in tumours and their adjacent non‐malignant tissues (TCGA). (C) Radar map of DCAF7 expression in normal and tumour tissues (TCGA/GTEx integrated data). Dot positions correspond to DCAF7 expression intensities. (D) Proteomic analysis of DCAF7 abundance in normal tissues and primary tumours using the Clinical Proteomic Tumor Analysis Consortium (CPTAC) dataset. (E) Representative immunohistochemistry from the Human Protein Atlas confirming tumour‐specific DCAF7 expression patterns. *p < .05, **p < .01, ***p < .001 and ns stood for no significant.

We first queried the Human Protein Atlas (HPA) to establish the baseline distribution of DCAF7 transcript in healthy tissues. The thymus, parathyroid gland, tonsil, lymph node and small intestine showed the highest mRNA abundance (Figure S1A). Protein‐level mapping revealed a partially discordant pattern (Figure S1B), consistent with post‐transcriptional regulation and antibody‐specific effects. Pan‐cancer analysis in Tumor Immune Estimation Resource 2.0 (TIMER2.0) demonstrated significant DCAF7 up‐regulation in bladder urothelial carcinoma (BLCA), breast invasive carcinoma (BRCA), cholangiocarcinoma (CHOL), colon adenocarcinoma (COAD), oesophageal carcinoma, head‐and‐neck squamous cell carcinoma (HNSC), LIHC, lung adenocarcinoma (LUAD), lung squamous cell carcinoma (LUSC), rectum adenocarcinoma, stomach adenocarcinoma (STAD) and uterine corpus endometrial carcinoma (UCEC) patients (Figure 1A). Matched‐pair comparisons confirmed similar trends (Figure 1B and Table S1). Because normal‐tissue representation in TCGA is limited for several entities, we merged TCGA tumours with GTEx normals. Except for tumour types lacking GTEx controls, DCAF7 was significantly higher in most cancers (Figure 1C and Table S2). CPTAC proteomics corroborated the transcript findings that DCAF7 protein was markedly increased in BRCA, COAD, LUAD, pancreatic adenocarcinoma (PAAD) and LIHC, but decreased in UCEC, HNSC and glioblastoma multiforme (GBM) (Figure 1D). Immunohistochemistry validated protein overexpression in selected tumours (Figure 1E), while subcellular localisation studies showed dual nucleoplasmic and cytosolic distribution in both A‐431 and U‐251 MG cells (Figure S1C,D). Collectively, DCAF7 is variably but frequently over‐expressed across malignancies and resides in both nuclear and cytosolic compartments.

### 2.2 DCAF7 expression stratifies survival and clinicopathological risk, with pronounced impact in LIHC

**FIGURE 2:**
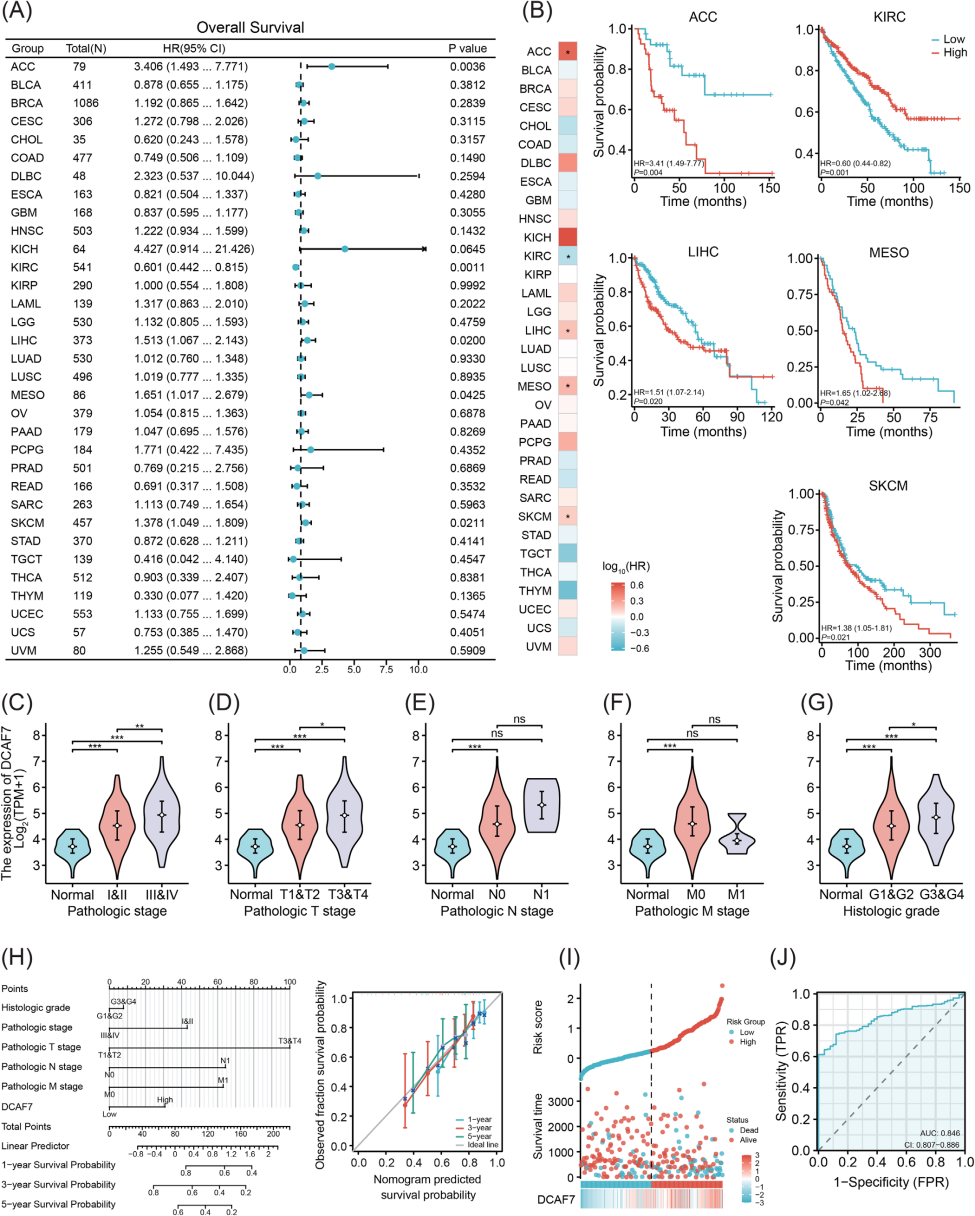
Prognostic significance of DCAF7 and clinical validation in LIHC. (A) Forest plot of univariate Cox analyses showing the effect of DCAF7 expression on overall survival (OS) across TCGA tumour types. (B) Kaplan–Meier curves comparing OS in high‐ versus low‐DCAF7 groups (median cut‐off) in selected cohorts. (C–G) Association of DCAF7 expression with LIHC clinicopathological parameters: overall pathological stage (C), T stage (D), N stage (E), M stage (F) and histological grade (G), assessed by Welch's one‐way ANOVA (*p < .05; **p < .01; ***p < .001; ns, not significant). (H) Nomogram combining DCAF7 and clinical variables to predict 1‐, 3‐ and 5‐year OS in LIHC; calibration curves (right) demonstrate agreement between predicted and observed survival. (I) TCGA‐LIHC risk model based on DCAF7: distribution of risk scores (top), survival status/time (middle) and *Z*‐score normalised gene‐expression heat map (bottom). (J) Receiver‐operating‐characteristic (ROC) curves depicting diagnostic performance of DCAF7 across cancers; area under the curve (AUC) indicates discrimination accuracy.

Comprehensive survival modelling (overall survival [OS], disease‐specific survival [DSS] and progression‐free interval [PFI]) is summarised in Figures 2A and S2A,C. For OS, high DCAF7 expression was associated with poorer outcomes in adrenocortical carcinoma (ACC), LIHC, mesothelioma (MESO) and skin cutaneous melanoma (SKCM) (*p* < .05; HR > 1). In contrast, it predicted improved OS in kidney renal clear cell carcinoma (KIRC) (*p* < .05; HR < 1) (Figure 2B). A similar pattern was evident for DSS: elevated DCAF7 correlated with shorter DSS in ACC and SKCM, yet remained a favourable marker in KIRC (Figure S2B). PFI analysis mirrored these findings – overexpression of DCAF7 portended earlier progression in ACC, cervical squamous cell carcinoma and endocervical adenocarcinoma (CESC), LIHC and uveal melanoma (UVM), while continuing to confer a protective effect in KIRC (Figure S2D). Given the consistent correlation between elevated DCAF7 expression and adverse clinical outcomes in LIHC, we selected LIHC as the primary cancer type to investigate DCAF7 functional role in tumour progression. High expression correlated with advanced TNM stage and poorer histological grade (Figure 2C–G) and remained an independent predictor on multivariable logistic regression (Table 1). A nomogram integrating DCAF7 and clinical factors accurately estimated 1‐, 3‐ and 5‐year survival, with calibration curves showing close agreement between predicted and observed probabilities (Figure 2H). Additionally, temporal calibration curves showed that the nomogram's predicted survival closely matched observed outcomes across all time points, supporting its clinical use in LIHC. A DCAF7‐based risk model (Table S3) further stratified patients, with the high‐risk group reaching endpoints sooner, underscoring DCAF7 as a marker of aggressive disease (Figure 2I). Receiver‐operating‐characteristic (ROC) analysis confirmed the strong discriminatory power of DCAF7 in diagnosing LIHC, yielding an area under the curve (AUC) of .846 in TCGA data (Figure 2J).

**TABLE 1:** Univariate and multivariate Cox regression analysis for overall survival in LIHC.

		Univariate analysis		Multivariate analysis	
Characteristic	Total (*N*)	Hazard ratio (95% CI)	*p* value	Hazard ratio (95% CI)	*p* value
Age	373				
≤60	177	Reference			
>60	196	1.205 (.850–1.708)	.295		
Gender	373				
Female	121	Reference			
Male	252	.793 (.557–1.130)	.200		
Pathologic T stage	370				
T1	183	Reference		Reference	
T2	94	1.431 (.902–2.268)	.128	1.456 (.805–2.634)	.214
T3	80	2.674 (1.761–4.060)	**<.001**	2.848 (1.697–4.781)	**<.001**
T4	13	5.386 (2.690–10.784)	**<.001**	5.083 (1.822–14.185)	**<.001**
Pathologic N stage	258				
N0	254	Reference			
N1	4	2.029 (.497–8.281)	.324		
Pathologic M stage	272				
M0	268	Reference		Reference	
M1	4	4.077 (1.281–12.973)	**<.005**	1.759 (.407–7.591)	.449
Histologic grade	368				
G1	55	Reference			
G2	178	1.162 (.686–1.969)	.576		
G3	123	1.185 (.683–2.057)	.545		
G4	12	1.681 (.621–4.549)	.307		
AFP (ng/mL)	279				
≤400	215	Reference			
>400	64	1.075 (.658–1.759)	.772		
DCAF7	373	1.354 (1.084–1.690)	**<.01**	1.371 (1.031–1.823)	**<.05**

Bold value expressing statistically significant *P* values using conventional thresholds (e.g., “.002” reported as “<.01” and “.017” reported as “<.05”, etc).

### 2.3 Landscape of DCAF7 genomic alterations and their clinical impact

**FIGURE 3:**
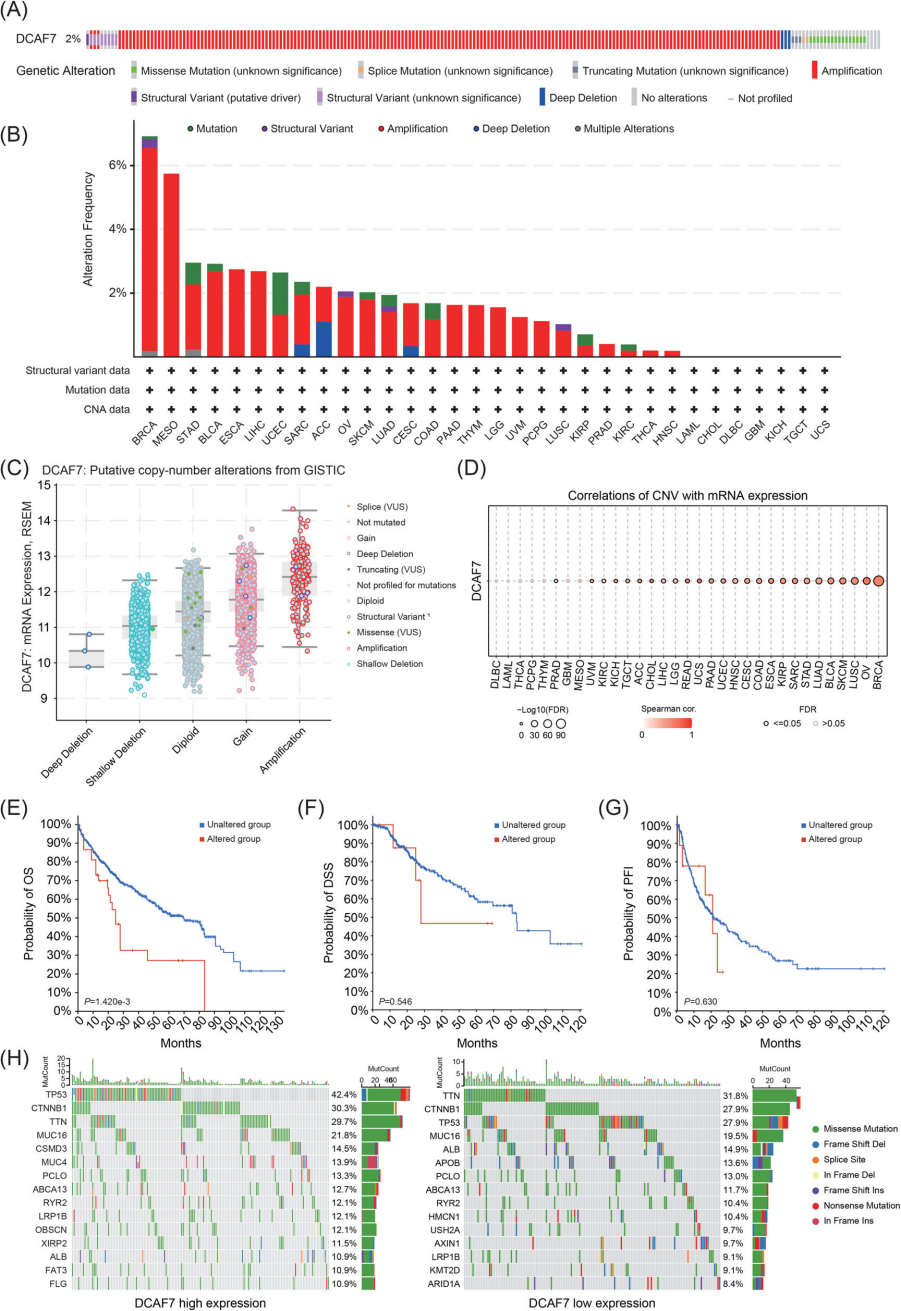
Genomic landscape of DCAF7 in human cancers. (A) cBioPortal overview of DCAF7 alteration frequency and mutation categories across TCGA tumours. (B) Bar plot of DCAF7 alteration rates by cancer type. (C) Box plot showing DCAF7 mRNA abundance stratified by copy‐number status (deep deletion, shallow deletion, diploid, gain, amplification). (D) Spearman correlation between copy‐number variation (CNV) and DCAF7 expression across cohorts in the GSCA database. (E–G) Kaplan–Meier curves for LIHC comparing overall survival (OS; E), disease‐specific survival (DSS; F) and progression‐free interval (PFI; G) in patients with versus without DCAF7 alterations. (H) Waterfall plots depicting the 15 most frequently mutated genes in LIHC samples with high versus low DCAF7 expression.

Somatic mutation is a major driver of oncogenesis.^15^ Analysis of 32 TCGA cohorts in cBioPortal showed DCAF7 alterations in ∼2% of tumours, spanning missense, splice‐site and truncating variants, amplifications, structural rearrangements and deep deletions (Figure 3A). Incidence varied by histology and exceeded 6% in breast cancer (Figure 3B). In LIHC, the dominant event was copy‐number amplification. Across cancers, DCAF7 copy number correlated positively with mRNA abundance, implicating gene dosage in transcriptional up‐regulation (Figure 3C). This association was strong in BRCA and ovarian serous cystadenocarcinoma (OV) but negligible in DLBC, LAML, THCA, PCPG and THYM (Figure 3D and Table S4), indicating additional regulatory controls in those entities. Besides, shallow deletions were common in most tumours except CHOL, KIRP, THCA and UVM (Figure S3A). Variant mapping highlighted several dispersed hotspots, with the frameshift L150Wfs*7 in exon 4 being most frequent (Figure S3B). Clinically, LIHC cases harbouring DCAF7 gene alterations had significantly shorter OS (*p* = 1.42 × 10^−^^3^), while DSS and PFI were unchanged (Figure 3E–G). Mutation‐spectrum comparison between high‐ and low‐DCAF7 LIHC tumours identified TP53, CTNNB1 and TTN as the most recurrent co‐mutated genes; missense substitutions constituted the largest variant class (Figure 3H).

### 2.4 Epigenetic regulation of DCAF7: DNA methylation and RNA modifications

#### 2.4.1 DNA methylation

**FIGURE 4:**
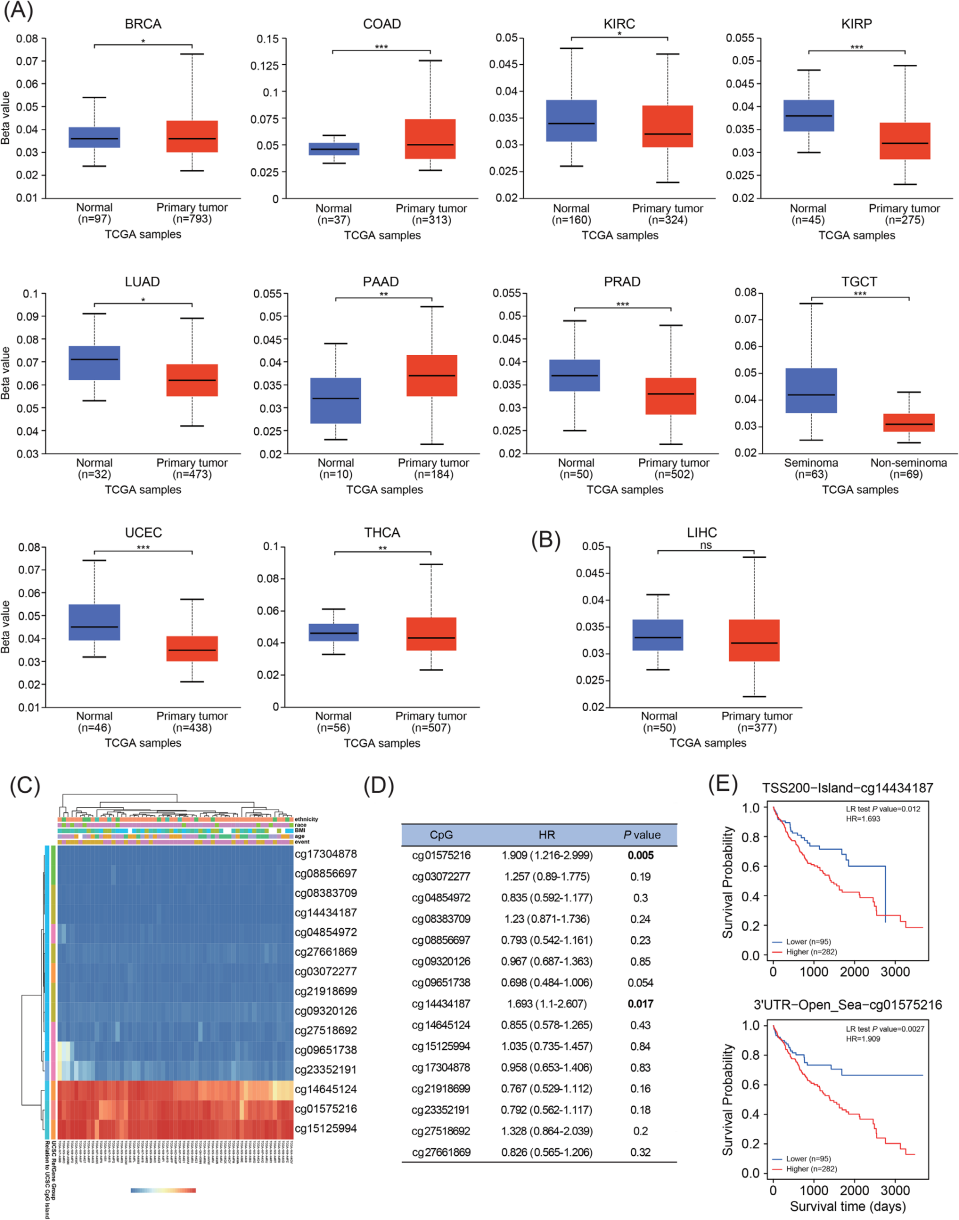
DNA methylation landscape of DCAF7. (A and B) Promoter β‐values of DCAF7 across cancers and matched normals queried in UALCAN; β ranges from 0 (unmethylated) to 1 (fully methylated). (C) MethSurv heat map correlating DCAF7 mRNA abundance with CpG‐site methylation. (D) Forest plot summarising the prognostic impact of DCAF7 methylation in LIHC. (E) Kaplan–Meier curves for LIHC stratified by methylation status at CpG loci cg01575216 and cg14434187. *p < .05, **p < .01, ***p < .001.

DNA methylation is a major epigenetic regulator of gene expression and tumour behaviour.^16^ UALCAN analysis revealed significant DCAF7 promoter hypomethylation in KIRC, KIRP, LUAD, prostate adenocarcinoma (PRAD), testicular germ‐cell tumour, UCEC and BLCA, whereas hypermethylation was evident in BRCA, COAD and PAAD relative to matched normal tissues (Figures 4A and S4). Promoter methylation in LIHC did not differ from normal liver (Figure 4B). MethSurv profiling of intragenic CpGs showed widespread hypomethylation across DCAF7 in LIHC (Figure 4C). Two specific CpG sites (cg01575216 and cg14434187) were prognostically informative as higher methylation at either locus was associated with reduced survival (Figure 4D,E and Table S5), suggesting that locus‐specific, rather than global, DNA methylation of DCAF7 modulates LIHC outcome.

#### 2.4.2 RNA modification

**FIGURE 5:**
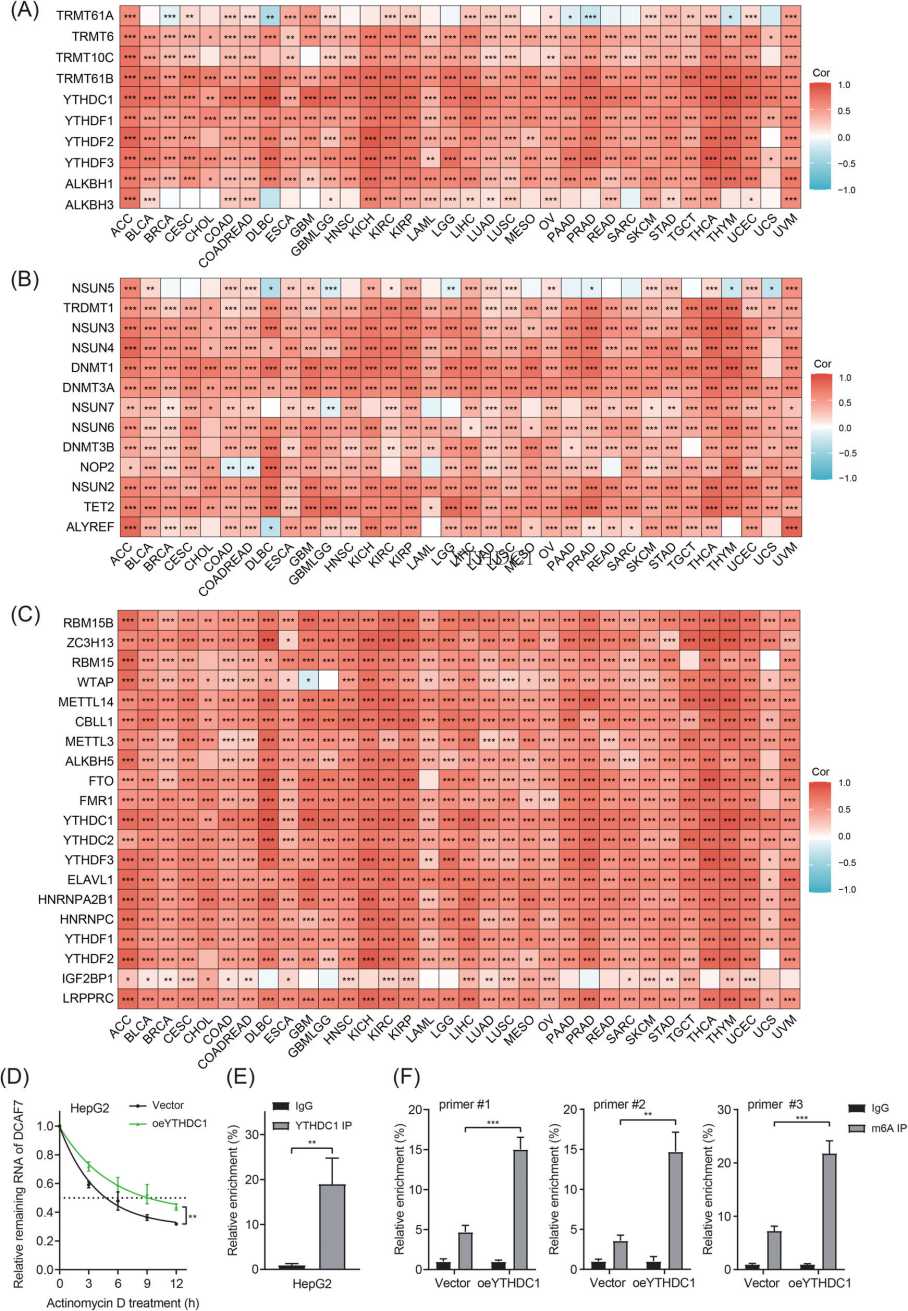
RNA modification landscape of DCAF7: YTHDC1 recognition of m^6^A sites promotes DCAF7 mRNA stability in LIHC. (A–C) Heat maps depicting correlations between DCAF7 expression and regulators of m^1^A (A), m^5^C (B) and m^6^A (C) across 33 TCGA tumour types. Values represent Pearson correlation coefficients. (D) HepG2 cells with YTHDC1 overexpression were treated with actinomycin D for 0–12 h, and the mRNA stability of DCAF7 was checked by qPCR. (E) The binding of YTHDC1 to DCAF7 mRNA in HepG2 cells was checked by RIP‐PCR using an anti‐YTHDC1 antibody. (F) m^6^A RIP‐qPCR analysis of DCAF7 mRNA in HepG2 cells transfected with control or DCAF7 expression plasmid. *p < .05, **p < .01, ***p < .001 (mean ± SD; Student's *t*‐test; *n* = 3).

mRNA modification is a critical layer of post‐transcriptional control with clear links to malignant transformation and progression.^17^ Among the >170 known chemical marks, m^1^A, m^5^C and m^6^A are the most extensively characterised, each regulated by dedicated ‘writer’, ‘eraser’ and ‘reader’ proteins.^18^ To explore whether RNA methylation influences DCAF7, we correlated its expression with 43 well‐defined regulators of m^1^A, m^5^C and m^6^A (Table S6).^19^ Across cancers, DCAF7 showed positive associations with the majority of writers, erasers and readers for all three marks (Figure 5A–C). In LIHC, the strongest correlation was with YTHDC1 (*r* = .806, *p* < .001), a dual m^1^A/m^6^A reader, implicating this protein as a potential upstream modulator. Consistent with this notion, overexpression of YTHDC1 in HepG2 cells markedly increased DCAF7 mRNA abundance and extended its half‐life, indicating enhanced transcript stability (Figures 5D and S5A,B). RNA immunoprecipitation (RIP)‐qPCR analysis demonstrated that YTHDC1 directly bound to DCAF7 mRNA (Figure 5E). Moreover, sequence‐based RNA adenosine methylation site predictor (SRAMP) analysis prediction identified four high‐confidence m^6^A sites within the DCAF7 transcript at positions 2385, 2393, 4145 and 5522 (Figure S5C and Table S7). Based on these predicted loci, we designed three primer sets to perform MeRIP assays and found elevated m^6^A enrichment in these regions following YTHDC1 overexpression (Figure 5F). Together, these data suggest that m^6^A deposition on DCAF7 mRNA and its recognition by YTHDC1 cooperatively enhance DCAF7 expression, thereby linking RNA methylation machinery to oncogenic signalling in LIHC and potentially other tumour types.

### 2.5 DCAF7 shapes the tumour immune microenvironment and aligns with immunotherapy biomarkers

**FIGURE 6:**
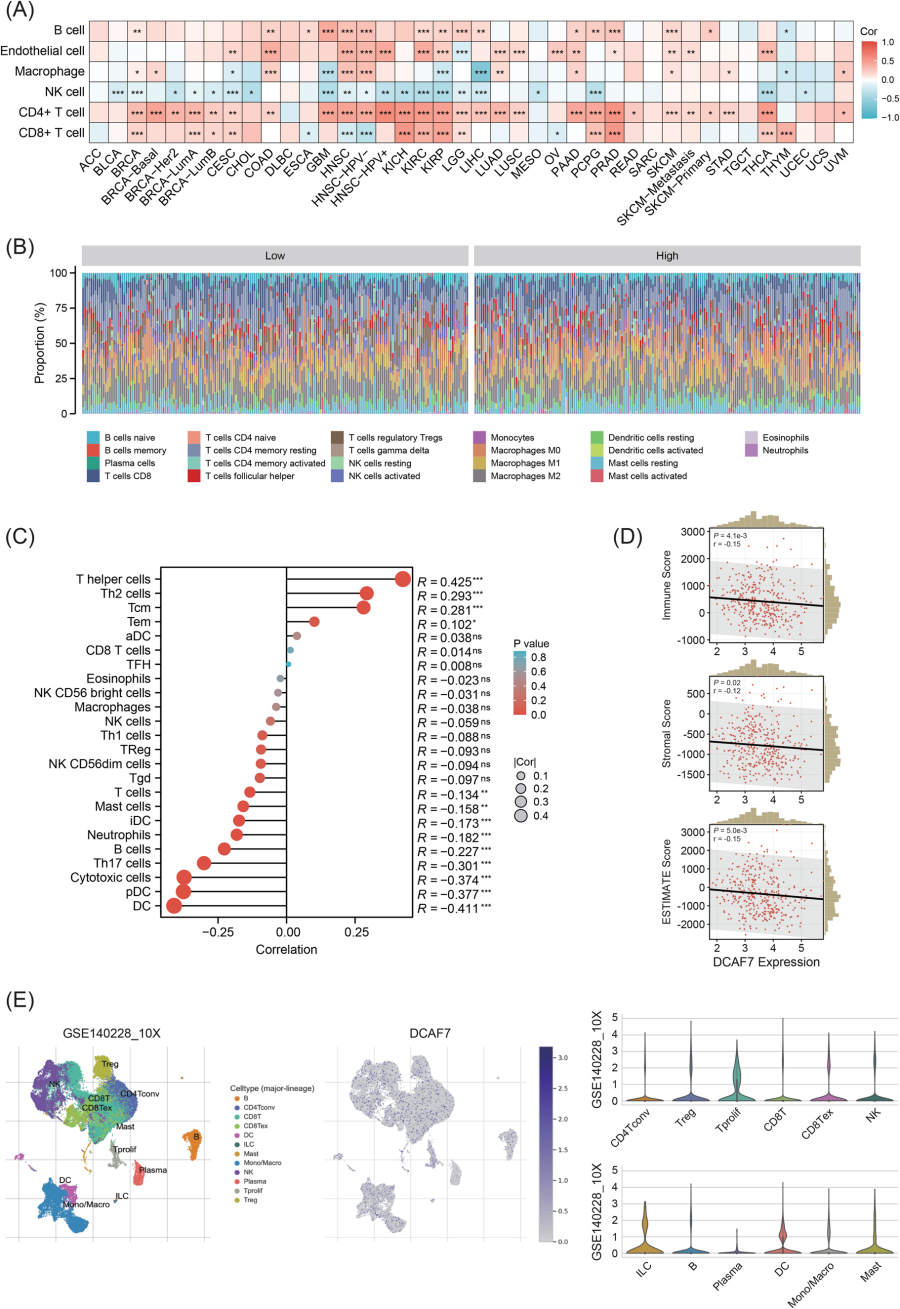
Association between DCAF7 expression and immune infiltration. (A) Heatmap of Spearman correlations between DCAF7 mRNA and infiltration of six immune lineages (B cells, endothelial cells, macrophages, NK cells, CD4⁺ T cells and CD8⁺ T cells) estimated by TIMER2.0 across 33 cancers. (B) Stacked bar chart of CIBERSORT‐derived fractions of 22 immune subsets in LIHC, stratified by median DCAF7 expression. (C) Lollipop plot showing Spearman correlations between DCAF7 and ssGSEA scores for 24 immune cell types in LIHC. (D) Scatter plots relating DCAF7 expression to ImmuneScore, StromalScore and ESTIMATEScore (ESTIMATE algorithm) in LIHC. (E) UMAP of single‐cell RNA‐seq data (GSE140228_10X) illustrating DCAF7 expression across LIHC cell populations, with violin plots detailing expression by cell type. *p < .05, **p < .01, ***p < .001; ns, not significant.

The tumour microenvironment (TME), comprising stromal fibroblasts, immune infiltrates, extracellular‐matrix components and soluble mediators, critically shapes diagnosis, prognosis and treatment response.^20^, ^21^ Using TIMER2.0, we correlated DCAF7 expression with six immune lineages across 33 TCGA tumours (Table S8). CD4⁺ T cell abundance showed the strongest positive association in 28 entities, whereas natural‐killer cells were inversely related in 20 (Figure 6A). In LIHC, CIBERSORT deconvolution generated a 22‐cell immune profile stratified by DCAF7 level (Figure 6B and Table S9). ssGSEA confirmed significant correlations with 24 immune subsets (Table S10); T helper, Th2, central memory (Tcm) and effector memory (Tem) T cells displayed the most pronounced positive links (Figure 6C). ESTIMATE scores indicated a modest but significant negative correlation between DCAF7 and overall Immune/Stromal/ESTIMATE content (Figure 6D). Single‐cell RNA‐seq from Tumor Immune Single‐cell Hub (TISCH) revealed preferential DCAF7 expression in proliferating T cells (T prolif) across three LIHC datasets (GSE140228_10X, GSE140228_Smartseq2 and GSE98638) (Figures 6E and S6A–C). We also performed HepG2–T cell co‐culture assay to verify the association between DCAF7 expression and CD8^+^ T cell cytotoxicity. Inverted microscopy indicated evident tumour cell lysis in the DCAF7‐knockout group, which became more pronounced with higher effector‐to‐target (E:T) ratios (Figure S6D). Consistently, enzyme‐linked immunosorbent assay (ELISA) analysis of the co‐culture supernatants showed a significant elevation of interferon‐γ (IFN‐γ) secretion following DCAF7 depletion (Figure S6E). These findings indicated that loss of DCAF7 enhances CD8⁺ T cell‐mediated cytotoxic activity against HepG2 cells.

**FIGURE 7:**
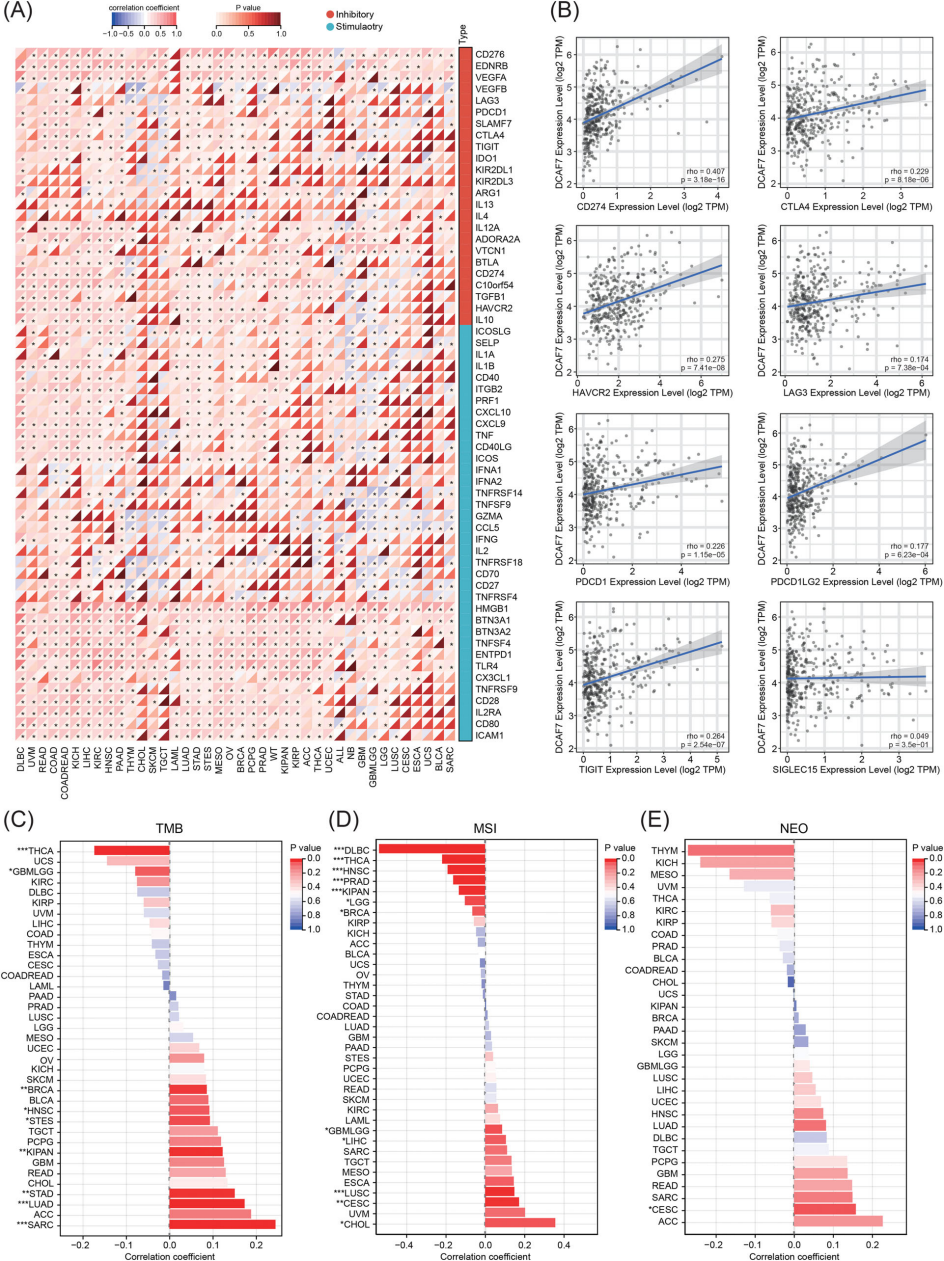
Links between DCAF7 expression and immunotherapy‐relevant biomarkers. (A) Heatmap of Spearman correlations between DCAF7 and 60 immune‐checkpoint genes across pan‐cancer. (B) Scatter plots illustrating correlations between DCAF7 and key checkpoints (CD274, CTLA4, HAVCR2, LAG3, PDCD1, PDCD1LG2, TIGIT and SIGLEC15) in LIHC through the TIMER2.0 database. (C–E) Bar graphs summarising Spearman correlation coefficients between DCAF7 and tumour mutational burden (TMB; C), microsatellite instability (MSI; D) and neo‐antigen load (NEO; E) in TCGA pan‐cancer cohorts. *p < .05, **p < .01, ***p < .001; ns, not significant.

Immune‐checkpoint (ICP) genes regulate immune infiltration and immunotherapy efficacy.^22^ DCAF7 expression correlated positively with most of 60 evaluated ICPs across cancers (Figure 7A). In LIHC, seven of eight key checkpoints (CD274, CTLA4, HAVCR2, LAG3, PDCD1, PDCD1LG2, TIGIT) rose in tandem with DCAF7, whereas SIGLEC15 did not (Figure 7B). Tumour mutational burden (TMB), microsatellite instability (MSI) and neoantigen load (NEO) are considered predictors for the response to tumour immunotherapy within the TME.^23^, ^24^, ^25^ We observed that high DCAF7 aligned with elevated TMB in LUAD, BRCA, stomach and oesophageal carcinoma (STES), sarcoma (SARC), pan‐kidney cohort (KIPAN), STAD and HNSC (Figure 7C and Table S11) and with increased MSI in glioma (GBMLGG), CESC, LUSC, LIHC and CHOL (Figure 7D and Table S12). DCAF7 also correlated with higher NEO in CESC (Figure 7E and Table S13). Collectively, these multi‐platform data indicate that DCAF7 shapes the immune milieu by influencing lymphocyte subsets, checkpoint‐gene expression and genomic‐instability metrics, thereby positioning it as a potential modulator of anti‐tumour immunity across cancers.

### 2.6 DCAF7‐centred interactome reveals druggable vulnerabilities and candidate therapeutics

**FIGURE 8:**
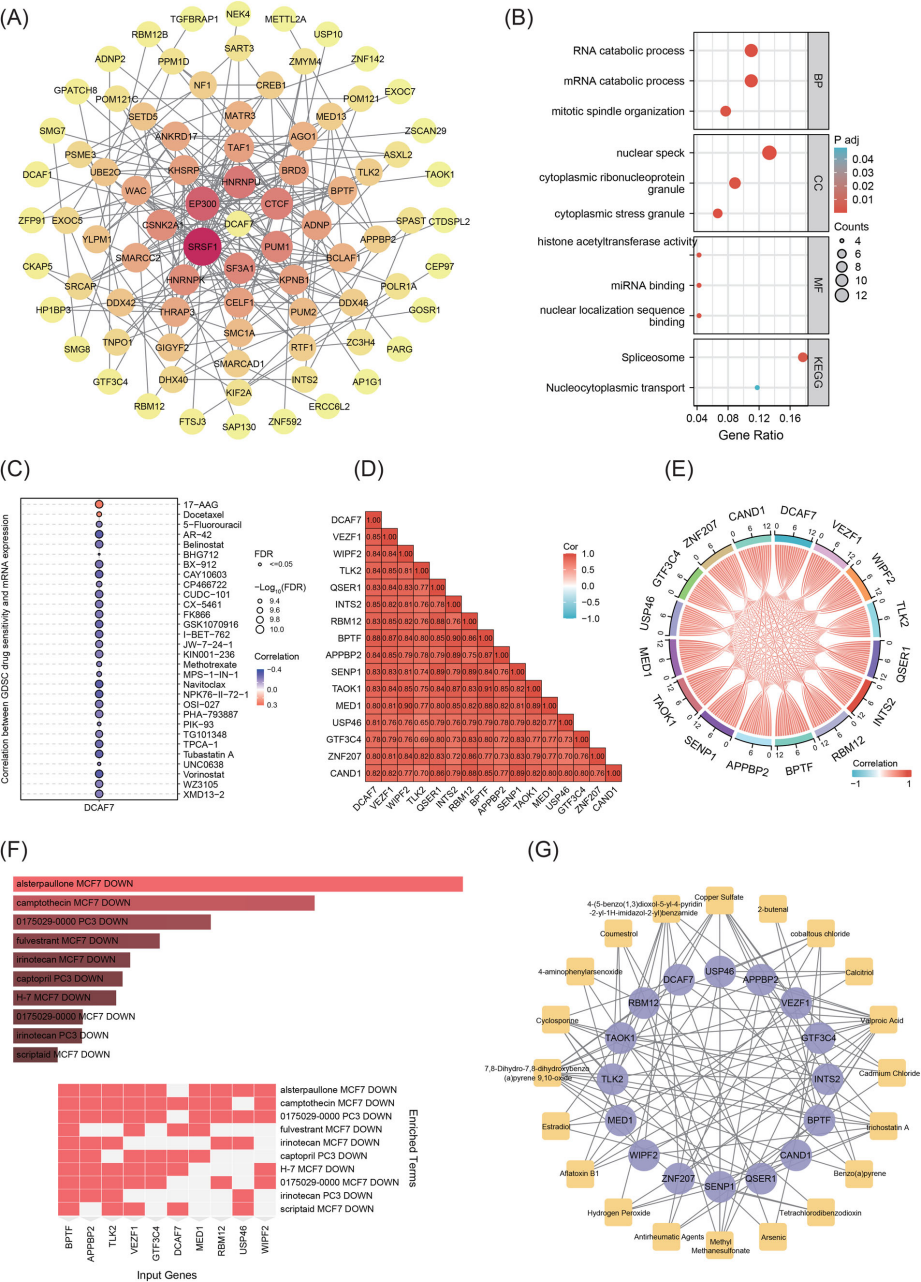
Co‐expression network of DCAF7 and in silico drug‐sensitivity prediction. (A) Cytoscape network of the top 100 genes co‐expressed with DCAF7 across pan‐cancer datasets; node size and colour intensity reflect degree centrality. (B) GO (BP, CC, MF) and KEGG enrichment of the co‐expressed gene set (adjusted *p* < .05). (C) Spearman correlations between DCAF7 mRNA and half‐maximal inhibitory concentration (IC_50_) values for GDSC compounds. (D and E) Pair‐wise correlations of the 15 genes most tightly linked to DCAF7 in LIHC visualised as a heat map (D) and chord diagram (E). (F) Predicted small‐molecule modulators of the LIHC co‐expression module identified through DSigDB (Enrichr). (G) Minimal protein–chemical interaction graph for the LIHC module; purple nodes, genes; yellow nodes, candidate compounds.

To map the oncogenic interactome surrounding DCAF7, we extracted the 100 transcripts most strongly co‐expressed with DCAF7 in TCGA using GEPIA2 and built a protein–protein interaction network in Cytoscape (Figure 8A and Table S14). Gene Ontology (GO) and Kyoto Encyclopedia of Genes and Genomes (KEGG) enrichment showed that this module is enriched for RNA catabolic process, mitotic spindle organisation, histone acetyltransferase activity, nuclear localisation sequence binding and spliceosome (Figure 8B and Table S15), consistent with roles in RNA processing and cell‐cycle control. Drug‐response modelling with the Genomics of Drug Sensitivity in Cancer (GDSC) dataset indicated that tumours with high DCAF7 expression are more sensitive to the HSP90 inhibitor 17‐AAG and to docetaxel; in fact, DCAF7 levels inversely correlated with IC_50_ values for >20 anti‐cancer agents (Figure 8C and Table S16), pointing to a broad influence on chemosensitivity. In LIHC, 15 hub genes most tightly correlated with DCAF7 displayed strong pairwise positive associations, underscoring a coherent regulatory network (Figure 8D,E). Drug‐repositioning analysis of these hubs (Enrichr/DSigDB) highlighted alsterpaullone as the top candidate compound (Figure 8F and Table 2). Complementary chemical‐gene interaction mapping in the Comparative Toxicogenomics Database (CTD) database identified several additional agents targeting multiple nodes of the network (Figure 8G and Table 3). The top five medications were valproic acid, 7,8‐dihydro‐7,8‐dihydroxybenzo(a)pyrene 9,10‐oxide, copper sulphate, cyclosporine and trichostatin A. Together, these data delineate a DCAF7‐centred module linked to RNA metabolism and mitotic regulation and nominate several small molecules for pre‐clinical evaluation in LIHC.

**TABLE 2:** Candidate drugs predicted using DSigDB.

Drugs	Adjusted *p* value	Combined score	Genes
Alsterpaullone MCF7 DOWN	1.22E−05	371.7	USP46; MED1; GTF3C4; APPBP2; WIPF2; TLK2; ZNF207; VEZF1; RBM12; BPTF
Camptothecin MCF7 DOWN	1.44E−04	227.5	MED1; GTF3C4; APPBP2; WIPF2; TLK2; VEZF1; RBM12; DCAF7; BPTF
0175029‐0000 PC3 DOWN	8.81E−04	134.8	USP46; MED1; GTF3C4; CAND1; APPBP2; WIPF2; TLK2; ZNF207; VEZF1; RBM12; BPTF
Fulvestrant MCF7 DOWN	.001965	379.3	MED1; VEZF1; DCAF7; BPTF
Irinotecan MCF7 DOWN	.002843	120.5	USP46; CAND1; APPBP2; TLK2; ZNF207; RBM12; BPTF
Captopril PC3 DOWN	.002843	139.2	MED1; GTF3C4; APPBP2; VEZF1; DCAF7; BPTF
H‐7 MCF7 DOWN	.002843	111.4	GTF3C4; APPBP2; WIPF2; TLK2; VEZF1; DCAF7; BPTF
0175029‐0000 MCF7 DOWN	.004584	91.9	GTF3C4; APPBP2; WIPF2; TLK2; VEZF1; RBM12; BPTF
Irinotecan PC3 DOWN	.004584	108.3	USP46; CAND1; APPBP2; TLK2; ZNF207; BPTF
Scriptaid MCF7 DOWN	.006929	117.8	USP46; TLK2; VEZF1; DCAF7; BPTF

**TABLE 3:** Suggested top 10 chemicals interacting with DCAF7‐related top 15 genes in LIHC.

Id	Label	Degree	Betweenness
D014635	Valproic acid	14	92.39
D015123	7,8‐Dihydro‐7,8‐dihydroxybenzo(a)pyrene 9,10‐oxide	12	64.21
D019327	Copper sulphate	9	31.51
D016572	Cyclosporine	8	25.72
C012589	Trichostatin A	8	23.08
C459179	4‐(5‐Benzo(1,3)dioxol‐5‐yl‐4‐pyridin‐2‐yl‐1H‐imidazol‐2‐yl)benzamide	8	22.29
D008741	Methyl methanesulphonate	6	17.1
C018021	Cobaltous chloride	6	14.07
D016604	Aflatoxin B1	5	8.77
D004958	Estradiol	4	8.55

### 2.7 Single‐cell phenotype mapping and bulk transcriptomics reveal proliferative programs driven by DCAF7

**FIGURE 9:**
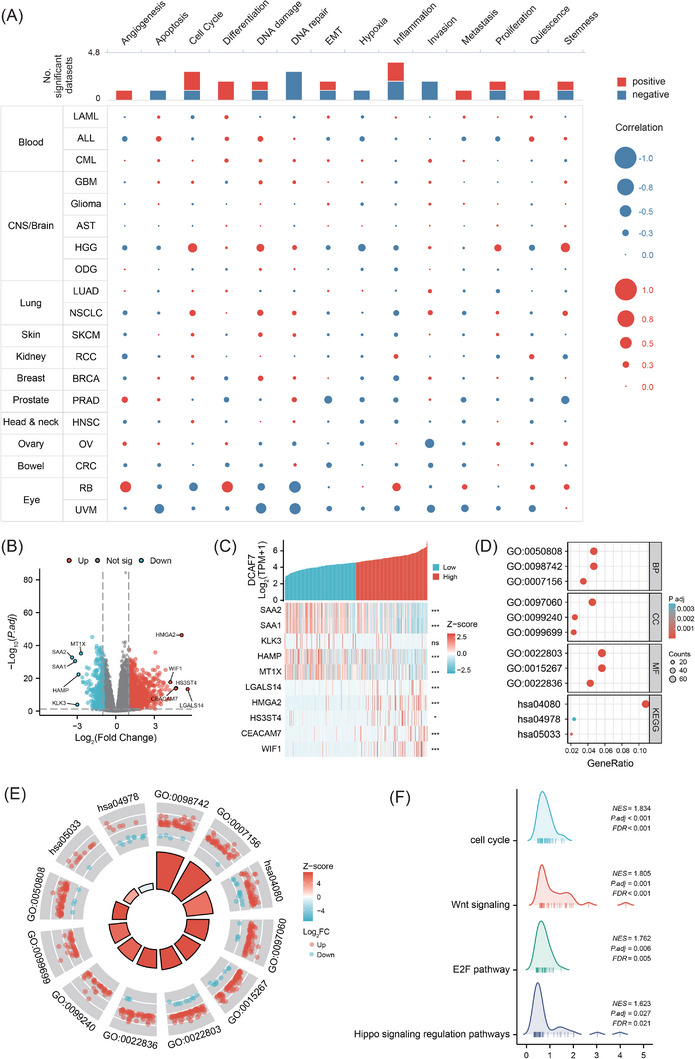
Differential gene expression and pathway enrichment associated with DCAF7. (A) CancerSEA single‐cell analysis linking DCAF7 expression to 14 functional states across 19 malignancies. (B) Volcano plot of differentially expressed genes (DEGs) between DCAF7‐high and ‐low LIHC groups (|log2FoldChange (log_2_FC)| > 1, adjusted *p* value < .05); red, up‐regulated; blue, down‐regulated. (C) Heatmap showing coordinated expression of DCAF7 with its five most down‐regulated (SAA2, SAA1, KLK3, HAMP, MT1X) and up‐regulated (LGALS14, HMGA2, HS3ST4, CEACAM7 and WIF1) DEGs. (D) Bubble plot of GO and KEGG terms enriched in DCAF7‐related DEGs. (E) Circular diagram illustrating GO and KEGG terms enriched among the DEGs. In the inner ring, bar height is inversely proportional to the adjusted *p* value, and bar colour reflects the corresponding *Z*‐score. The outer ring lists protein‐coding genes associated with each term; bar height denotes log_2_FC, with positive values (up‐regulation) in red and negative values (down‐regulation) in blue. (F) GSEA ‘mountain’ plot highlighting pathways (e.g., Wnt, Hippo, E2F, cell‐cycle) activated in DCAF7‐high LIHC tumours.

Single‐cell interrogation with CancerSEA showed that DCAF7 expression is positively associated with cell cycle progression and differentiation, but negatively linked to DNA repair and invasive signatures across aggregated tumour data (Figure 9A). When analysed by tumour type, distinct patterns emerged. In LAML, DCAF7 expression positively correlated with differentiation, inflammation, quiescence, proliferation, metastasis and epithelial–mesenchymal transition (EMT). In high‐grade glioma (HGG), it tracked with stemness, cell cycle and DNA‐damage accumulation. A similar proliferative link was evident in LUAD, where DCAF7 strongly aligned with cell cycle activation. By contrast, inverse associations were observed for inflammation in BRCA, stemness or EMT in PRAD, invasion in OV and multiple stress‐response pathways including DNA repair, DNA damage response, apoptosis and invasion in UVM (Figure S7A). Focusing on LIHC, stratification of TCGA tumours by DCAF7 level identified 1612 differentially expressed genes (DEGs), of which 1244 were up‐regulated and 368 down‐regulated in the high‐expression group (Figure 9B). Heatmap analysis delineated that the top‐ranked transcripts (SAA2, SAA1, KLK3, HAMP, MT1X, LGALS14, HMGA2, HS3ST4, CEACAM7 and WIF1) exhibited strong co‐expression with DCAF7 (Figure 9C). GO/KEGG enrichment revealed that DCAF7‐regulated genes are concentrated in synapse organisation, homophilic cell‐adhesion, gated channel activity and neuroactive ligand–receptor interaction, with mineral‐absorption pathways being the only category down‐regulated (Figures 9D andS7B). All other terms showed significant up‐regulation in the high‐risk subgroup (*Z*‐score > 1; Figure 9E and Table S17). Gene‐set enrichment analysis (GSEA) further demonstrated activation of cell‐cycle, Wnt, E2F and Hippo signalling in DCAF7‐amplified tumours (Figure 9F and Table S18). Taken together, these data indicate that DCAF7 promotes a proliferative, adhesion‐competent transcriptional program, particularly in LIHC, while variably modulating lineage‐specific functional states across cancers.

### 2.8 DCAF7 promotes LIHC cell growth and motility through canonical Wnt/β‐catenin activation

**FIGURE 10:**
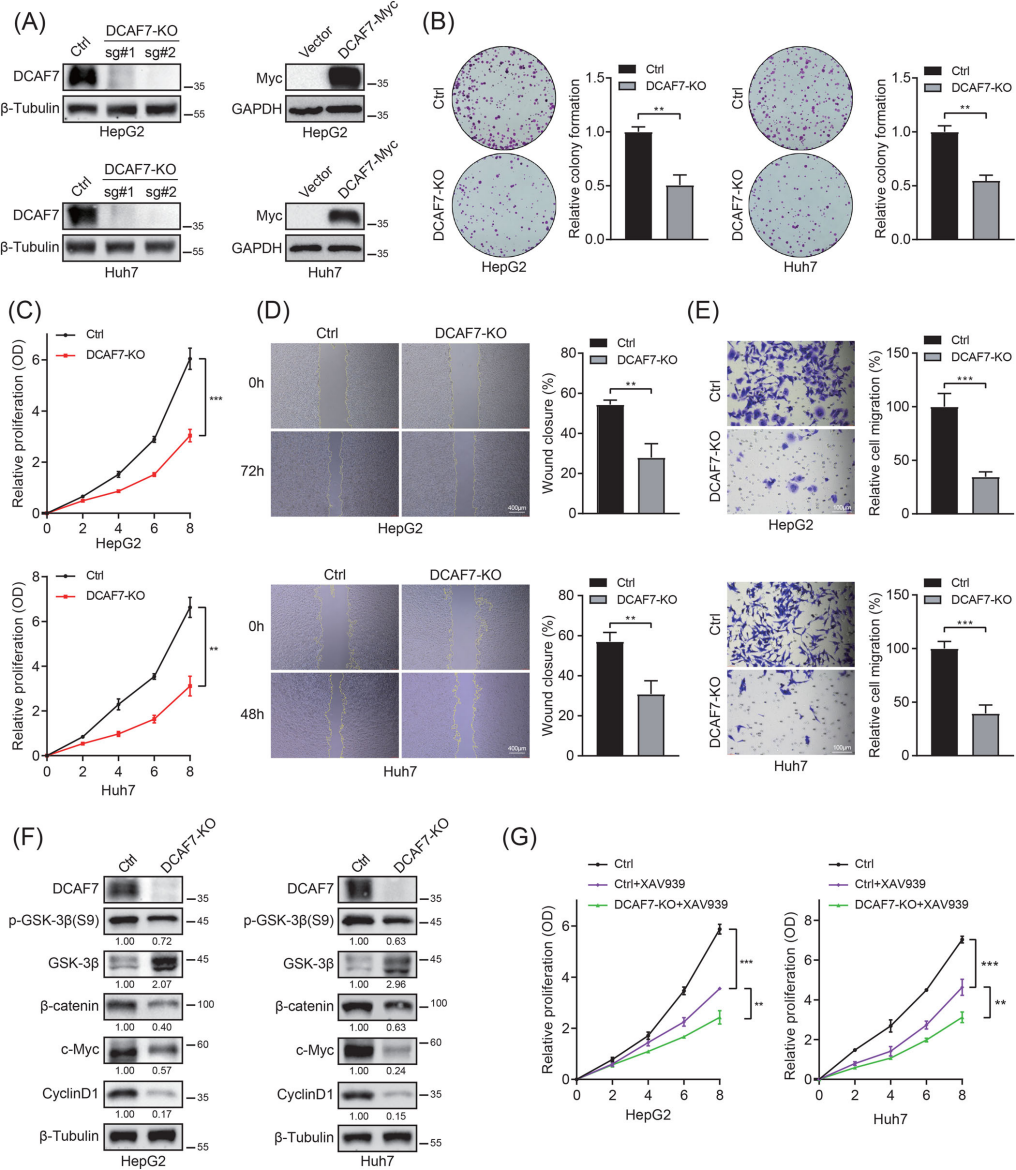
DCAF7 depletion attenuates Wnt/β‐catenin signalling and suppresses LIHC cell growth and migration. (A) Western blot was employed to quantify the knockout (left) and overexpression (right) efficiencies of DCAF7 in HepG2 and Huh7 cells. (B) Colony formation assay comparing Ctrl and DCAF7‐knockout (DCAF7‐KO) HepG2/Huh7 derivatives. (C) Growth curves showing reduced proliferation upon DCAF7 knockout; viability measured every other day using MTS assay. (D and E) Wound‐healing (D; scale bar = 400 µm) and transwell‐migration (E; scale bar = 100 µm) assays demonstrating impaired motility in DCAF7‐depleted cells. (F) Western blot of GSK‐3β, phospho‐GSK‐3β (Ser9), β‐catenin, c‐Myc and cyclin D1 following DCAF7 silencing. (G) Growth curves illustrating the effect of XAV939 (10 µM) alone or combined with DCAF7‐KO on LIHC cell proliferation. Data are mean ± SD of three independent experiments. *p < .05, **p < .01, ***p < .001.

To elucidate the functional contribution of DCAF7 to LIHC, we established stable HepG2 and Huh7 cell lines with either DCAF7 overexpression or CRISPR–Cas9‐mediated knockout using two independent sgRNAs (sg#1 and sg#2). Immunoblot analysis confirmed effective modulation of DCAF7 protein expression, and sg#1, which achieved complete knockout efficiency, was selected for subsequent experiments (Figure 10A). Loss of DCAF7 markedly reduced colony formation, whereas ectopic expression enhanced clonogenicity (Figures 10B and S8A). Consistent results were obtained with growth‐curve analysis: where knockout of DCAF7 curtailed proliferation, while its overexpression accelerated cell growth (Figures 10C and S8B). Scratch‐wound and transwell assays further showed that DCAF7 promotes motility, as overexpressing cells displayed increased migration, whereas DCAF7 knockout markedly suppressed this behaviour (Figures 10D,E and S8C,D). These assays indicate that DCAF7 acts as a pro‐tumourigenic driver in LIHC cells.

Mechanistically, western blot revealed that DCAF7 modulates the canonical Wnt cascade. In this pathway, β‐catenin stability is controlled by the Axin–APC destruction complex, with GSK‐3β acting as a central negative regulator whose activity is suppressed by phosphorylation at Ser9.^26^ Silencing the DCAF7 gene increased total GSK‐3β and diminished its inhibitory Ser9 phosphorylation, thereby activating the destruction complex and promoting β‐catenin degradation; downstream targets c‐Myc and cyclin D1 fell concordantly (Figure 10F). The opposite pattern – decreased active GSK‐3β, β‐catenin accumulation and target‐gene induction – was observed upon DCAF7 overexpression (Figure S8E). To further elucidate how DCAF7 engages the Wnt/β‐catenin signalling pathway, co‐immunoprecipitation (Co‐IP) assays were performed in HepG2 cells. Exogenous Co‐IP experiments using Myc‐tagged DCAF7 demonstrated that DCAF7 physically associates with both GSK‐3β and β‐catenin (Figure S8F). Consistently, endogenous Co‐IP confirmed the interaction between GSK‐3β and DCAF7 at the native protein level (Figure S8G). These results collectively indicated that DCAF7 forms a complex with the core components of Wnt cascade, thereby serving as a positive regulator of β‐catenin signalling. Functionally, DCAF7 knockout cooperated with the tankyrase inhibitor XAV939 (which destabilises β‐catenin) to further suppress LIHC‐cell growth (Figure 10G), indicating that concurrent targeting of DCAF7 and Wnt signalling may offer therapeutic benefit. To assess whether the oncogenic role of DCAF7 extends beyond LIHC, we focused on breast cancer, in which DCAF7 is highly expressed (Figure 1) and explored its biological effects in the BT549 cell line. DCAF7 overexpression significantly enhanced colony formation and accelerated cell growth, as indicated by growth‐curve analysis (Figure S8H–J). Consistently, scratch‐wound and transwell assays demonstrated that DCAF7 promoted cell migration in BT549 cells (Figure S8K,L). These findings indicate that DCAF7 enhances proliferation, motility and migration in breast cancer cells, supporting a broader, pan‐cancer role for DCAF7 in promoting tumour progression.

## 3 DISCUSSION

**FIGURE 11:**
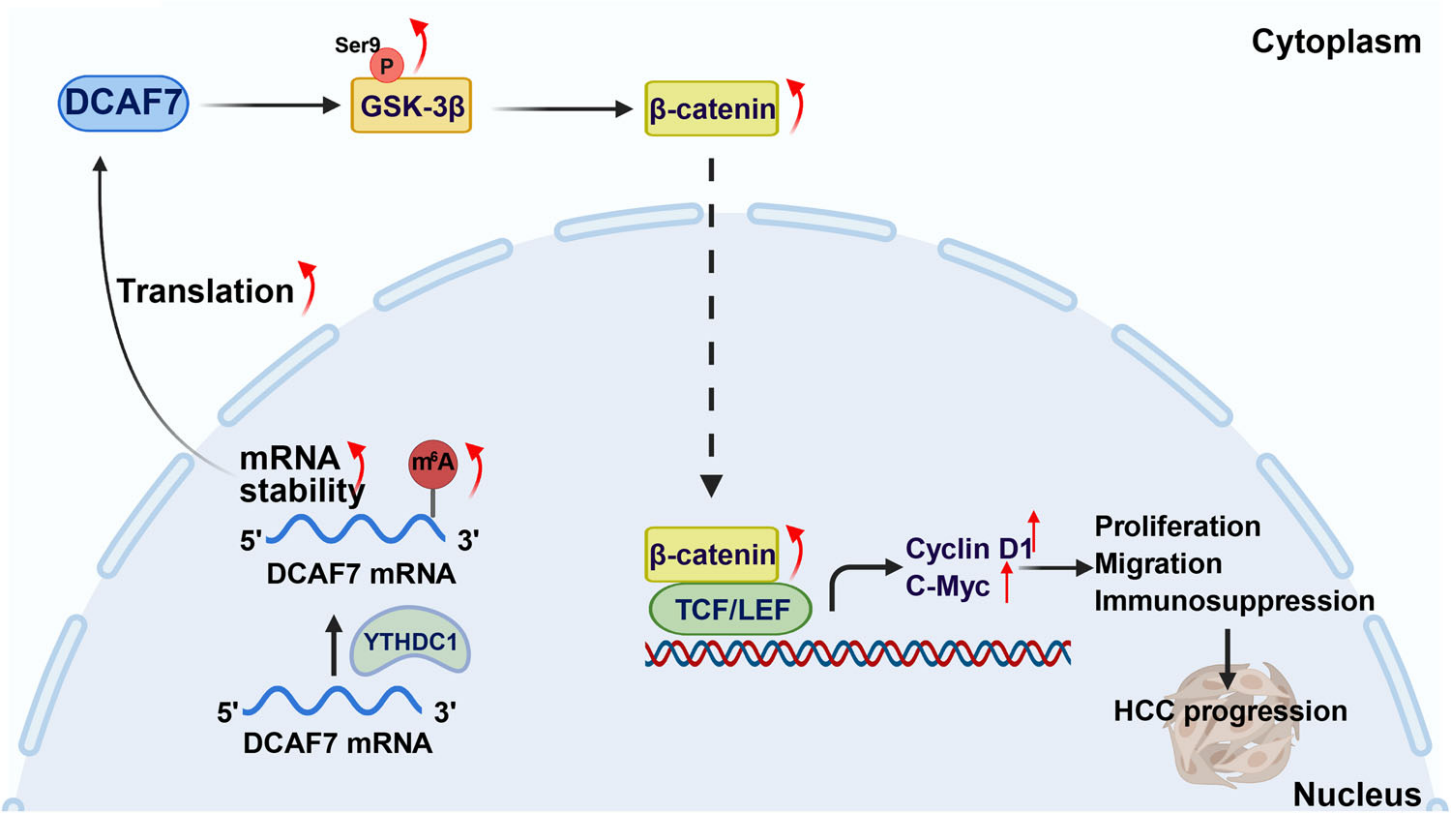
The schematic model illustrating the functional role of YTHDC1/m^6^A–DCAF7 axis in facilitating LIHC progression through activation of the Wnt/β‐catenin signalling pathway.

DCAF7 is an evolutionarily conserved adaptor protein whose biological importance has been documented from plants to mammals. Initially characterised for controlling anthocyanin biosynthesis in *Petunia* flowers,^27^ it subsequently emerged as a determinant of craniofacial development in zebrafish through modulation of the Edn1 pathway,^28^ of wing‐vein patterning and jump‐muscle maturation in *Drosophila* via its orthologue Wap,^29^ and of murine skin morphogenesis by repressing Hedgehog‐effector GLI1.^30^ Mechanistically, DCAF7 serves as a scaffold for multiple kinases: it restrains MEKK1, DYRK1 and HIPK2 to dampen osmotic‐stress signalling,^31^ co‐operates with DYRK1A at myogenic loci to promote myotube differentiation^32^ and bridges IRS1 with PI3K, enhancing AKT activation and cell proliferation.^33^ Although preliminary reports hinted at tumour‐related roles, its oncogenic functions and underlying mechanisms have remained largely undefined. By integrating multi‐omics resources (TCGA, GTEx, CPTAC, UALCAN, cBioPortal, CTD, CancerSEA and others), we provide the first pan‐cancer framework for its regulation and function. Our results show that DNA‐methylation changes and copy‐number variations jointly drive aberrant DCAF7 expression, which is tightly linked to survival across tumour types. Single‐cell and bulk immune‐deconvolution analyses demonstrate that DCAF7 reshapes the tumour immune microenvironment, altering T cell subsets and immune‐checkpoint expression. Mechanistic interrogation in LIHC further reveals that YTHDC1 enhances m^6^A methylation of DCAF7 mRNA, leading to increased DCAF7 expression, which in turn amplifies Wnt/β‐catenin signalling and promotes tumour proliferation and migration. Together, these findings establish DCAF7 as both a prognostic biomarker and a putative therapeutic target (Figure 11).

Our pan‐cancer analysis revealed tissue‐specific expression patterns of DCAF7, with significant down‐regulation in KICH at the RNA level compared with corresponding normal tissues (Figure 1B), and in UCEC, HNSC and GBM at the protein level (Figure 1D). These observations suggest that DCAF7 may exert context‐dependent roles in tumourigenesis, potentially functioning as a tumour suppressor in certain cancer types while acting as an oncogene in LIHC. Such dual functionality is not uncommon in cancer biology and has been reported for other regulatory proteins.^34^, ^35^ The underlying mechanisms of this differential expression may involve tissue‐specific epigenetic regulation, post‐transcriptional control (e.g., m^6^A modification, miRNAs, alternative splicing) and proteostasis, as well as potential effects of tumour purity and cellular composition. Elucidating the precise molecular basis of these expression differences is therefore essential for understanding the functional consequences of DCAF7 dysregulation across cancer contexts.

Building on these expression‐level insights, it is important to recognise that cancer progression itself arises from the dynamic interaction of genetic lesions, epigenetic re‐programming and plastic cell states that confer fitness under selective pressures.^36^ Driver events – loss‐of‐function alterations in tumour suppressors or gain‐of‐function changes in oncogenes – supply the initial proliferative and survival advantages, while ongoing genomic instability amplifies mutational diversity and intratumour heterogeneity.^37^ Within this framework, we found that DCAF7 is mutated in most malignancies, with the highest alteration rate in breast cancer. Although the overall frequency is modest (∼2%), copy‐number gains and deletions were observed and, in LIHC, independently predicted inferior OS. These data implicate structural variation in DCAF7 as a clinically relevant driver and support its use as a prognostic biomarker.

Epigenetic mechanisms further modulate DCAF7 activity. Aberrant DNA methylation, histone‐code disruption and chromatin remodelling can reprogram oncogenic transcription without changing the underlying DNA sequence.^38^ Consistent with this paradigm, we observed widespread promoter hypomethylation of DCAF7 across cancers, whereas two CpG islands in LIHC (cg01575216 and cg14434187) were hypermethylated and independently associated with poor outcome, underscoring the importance of locus‐specific rather than global methylation states.^39^ Beyond DNA, RNA chemistry adds an additional regulatory tier: DCAF7 mRNA levels correlated positively with multiple modifiers of m^1^A, m^5^C and m^6^A across tumours (Figure 5A–C). In LIHC, the strongest correlation was observed with YTHDC1, a dual m^1^A/m^6^A reader, suggesting it as a potential upstream modulator. We further experimentally validated that YTHDC1 enhances m^6^A methylation at four high‐confidence sites within DCAF7 mRNA, thereby stabilising DCAF7 transcripts and up‐regulating their expression. This convergence of genetic lesions, DNA‐ and RNA‐level epigenetic alterations strongly suggests that diverse oncogenic pressures co‐opt DCAF7 to support malignant growth and highlights its potential utility as an integrated genomic–epigenomic biomarker across cancer types.

The TME is a decisive determinant of both responsiveness and resistance to immune‐checkpoint blockade.^40^ Complex bidirectional signalling between malignant and immune cells establishes an immunosuppressive niche via checkpoints, cytokines and other soluble mediators.^41^ Accumulating evidence indicates that the transcriptional landscape of key immune‐regulatory genes conditions the efficacy of checkpoint inhibitors, underscoring the need to dissect tumour–immune crosstalk at the molecular level to refine biomarkers and rational combination regimens.^42^ In our analysis, DCAF7 expression showed robust positive associations with the infiltration of multiple immune subsets – including B cells, endothelial cells, macrophages, NK cells and both CD4⁺ and CD8⁺ T lymphocytes – across cancers. Paradoxically, higher DCAF7 corresponded to slightly lower composite Immune, Stromal and ESTIMATE scores, hinting that it may skew the balance toward an immunosuppressive milieu despite increased cellular influx. In LIHC, DCAF7 correlated strongly with PD‐1/PD‐L1 and CTLA‐4 expression, implying that tumours with elevated DCAF7 might preferentially exploit checkpoint pathways for immune evasion and could therefore benefit from dual targeting of DCAF7‐driven signalling and checkpoint blockade. Moreover, positive associations between DCAF7 and TMB, MSI or NEO in several cancers suggest that DCAF7 may also influence the antigenic landscape, further modulating anti‐tumour immunity and potential treatment response. Consistent with these bioinformatic predictions, our HepG2–T cell co‐culture assays demonstrated that DCAF7 knockout markedly enhanced CD8⁺ T cell‐mediated cytotoxicity and IFN‐γ secretion, providing functional evidence that DCAF7 restrains anti‐tumour immune activity within the TME.

Analysis of GDSC profiles revealed that tumours with high DCAF7 expression tend to be more responsive to several cytotoxic and targeted agents, indicating that DCAF7 could serve as a predictive marker of chemotherapy sensitivity. Network expansion in LIHC identified 15 tightly co‐expressed hub genes, and drug–gene mapping (DSigDB) prioritised alsterpaullone – a cyclin‐dependent‐kinase and GSK‐3 inhibitor – as the compound with the greatest network connectivity, nominating it for further pre‐clinical testing against DCAF7‐driven disease.

Although our multi‐omics data link DCAF7 to aggressive phenotypes, the downstream effector programs remain only partly defined. Integrative enrichment showed preferential involvement in passive transmembrane transport, ion‐channel regulation and neuroactive ligand–receptor interactions, while GSEA connected high DCAF7 to Wnt, Hippo, E2F and cell‐cycle pathways. These signatures suggested that DCAF7 promotes LIHC by accelerating proliferation and rewiring oncogenic signalling, with Wnt/β‐catenin emerging as the dominant axis. Canonically, Wnt ligand binding blocks the APC–Axin–GSK‐3β destruction complex, allowing β‐catenin to accumulate, translocate to the nucleus and drive genes such as c‐Myc and cyclin D1.^43^ Activating *CTNNB1* (encoding β‐catenin) mutations hard‐wire this circuitry in a sizeable subset of LIHCs, sustaining stemness, immune evasion and therapeutic resistance.^44^ Our gain‐ and loss‐of‐function experiments confirm that DCAF7 potentiates this pathway: overexpression increased β‐catenin and inhibitory Ser9 phosphorylation of GSK‐3β, whereas knockout had the opposite effect. To contextualise DCAF7 within canonical Wnt architecture, it is instructive to contrast it with AXIN1, the core scaffold of the β‐catenin destruction complex that recruits β‐catenin, GSK‐3β and APC to promote β‐catenin turnover.^45^ In LIHC, DCAF7 functions as a positive Wnt regulator: it elevates the inhibitory Ser9 phosphorylation of GSK‐3β, stabilises β‐catenin and induces c‐Myc/cyclin D1, a mechanism distinct from AXIN1's degradative role. Moreover, whereas AXIN1 is tightly controlled by tankyrase‐mediated PARsylation and ubiquitination,^46^ DCAF7 up‐regulation tracks with copy‐number gain and epigenetic/RNA‐modification changes in our pan‐cancer analysis. These differences argue that DCAF7 represents a mechanistically distinct, potentially more selective node for therapeutic Wnt modulation in LIHC. Accordingly, DCAF7 enhanced HepG2 and Huh7 proliferation and migration, while its depletion suppressed these traits. Pharmacological blockade of Wnt signalling with XAV939 blunted DCAF7‐mediated growth, firmly establishing Wnt/β‐catenin activation as the key downstream mechanism. Together, these findings position DCAF7 as both a mechanistic amplifier of Wnt signalling in hepatocarcinogenesis and a potential biomarker for selecting patients who might benefit from Wnt‐targeted combinations such as alsterpaullone.

Although our multi‐omics survey delivers a broad view of DCAF7 dysregulation, several caveats warrant consideration. Validation experiments were confined to hepatocellular carcinoma; whether the same molecular circuits operate in other DCAF7‐high tumours remains to be tested in lineage‐appropriate cell lines, organoids and animal models. Mechanistically, we demonstrated that DCAF7 amplifies Wnt/β‐catenin output, but the precise biochemical step – direct stabilisation of β‐catenin, modulation of the destruction complex or interaction with upstream receptors – has not been delineated. Dissecting these possibilities will require proteomic mapping, mutational epistasis and real‐time imaging of β‐catenin dynamics. In addition, our conclusions are drawn largely from bulk transcriptomic datasets, which average signals across malignant and stromal compartments; single‐cell and spatial transcriptomics will be essential to resolve cell‐type–specific effects of DCAF7 within the TME. Finally, given DCAF7's pleiotropic functions in immune regulation and developmental programs, as well as its comparatively high baseline expression in the thymus and small intestine (Figure S2A), systemic inhibition may carry risks of on‐target toxicity. Therefore, future therapeutic strategies should emphasise mechanism‐selective modulation – for instance, small molecules or targeted protein degraders that disrupt oncogenic interfaces within the Wnt/CRL4 adaptor complex – while integrating tumour‐selective delivery approaches such as hepatotropic GalNAc–siRNA, liver‐tropic lipid nanoparticles or loco‐regional administration for LIHC. For clinical translation, reversible modalities (e.g., RNAi/ASO or small molecules) are preferable, whereas CRISPR/Cas systems should remain confined to preclinical validation due to irreversible editing and delivery limitations in solid tumours. To comprehensively evaluate safety, human organoid assays (intestinal, thymic and hepatic) and conditional mouse models (Villin‐Cre, Foxn1‐Cre, Albumin‐Cre) should be employed, alongside pharmacodynamic monitoring of Wnt activity (nuclear β‐catenin, c‐Myc/cyclin D1). Collectively, these strategies aim to define the therapeutic window, minimise off‐target effects and enhance both the specificity and safety of DCAF7‐targeted interventions. Prospective, multi‐centre cohorts are also needed to confirm the biomarker value of DCAF7 and to evaluate its utility in stratifying patients for Wnt‐targeted or immunotherapeutic regimens.

In conclusion, this study positions DCAF7 as a multi‐layered oncogenic driver that integrates genetic, epigenetic and immune cues across cancers, with particularly strong evidence of Wnt‐mediated tumour‐promoting activity in hepatocellular carcinoma. By linking DCAF7 to immune‐checkpoint expression, genomic instability metrics and drug‐sensitivity profiles, we provide a framework for exploiting this molecule as both a prognostic indicator and a therapeutic target. These insights lay the groundwork for future mechanistic and translational investigations aimed at harnessing DCAF7 in precision oncology.

## 4 MATERIALS AND METHODS

### 4.1 Data acquisition and processing

The transcriptomic data of normal and tumour samples were sourced from TCGA database (https://portal.gdc.cancer.gov/), the GTEx database (http://commonfund.nih.gov/GTEx/) and the UCSC Xena database (https://xena.ucsc.edu/).^47^ The gene expression matrix in TPM format was obtained by converting the original read count values. To facilitate comparison among samples, RNA‐seq data were processed uniformly using the Toil process^48^ and subsequently log2(TPM+1)‐transformed for downstream analyses.

### 4.2 DCAF7 expression and subcellular localisation analysis

The differential expression patterns of the DCAF7 gene across various cancers were acquired via the ‘Gene_DE’ module in the TIMER2.0 database (http://timer.cistrome.org/).^49^ To verify DCAF7 expression in tumours and their paired adjacent normal tissues, the TCGA cancer types devoid of matching normal samples were excluded. Statistical evaluation was performed using the Wilcoxon signed rank test, and significant outcomes were defined at *p* value < .05. Besides, a comprehensive pan‐cancer analysis was conducted by extracting the TCGA data alongside the corresponding normal tissue data from the GTEx cohort. The results were visualised with the ‘ggplot2’ package of R software. DCAF7 protein level differences were obtained from the CPTAC available on the UALCAN website (https://ualcan.path.uab.edu/analysis‐prot.html)^50^ and compared with unpaired Student's *t*‐test. DCAF7 immunohistochemical and immunofluorescence staining images were retrieved from the HPA database (https://www.proteinatlas.org)^51^ to illustrate tissue distribution and subcellular localisation.

### 4.3 Survival, clinical and diagnostic analyses

Cox regression analyses and KM curves were adopted to assess the OS, DSS and PFI in pan‐cancer. The ‘survival’ package was used to fit the Cox proportional hazards regression models on the prognostic variables,^52^ and the results were visualised with the ‘survminer’ package and ‘ggplot2’ package. For survival map, cohorts with high and low DCAF7 expression levels were separated by median values. To assess the clinical relevance of DCAF7 in LIHC, we correlated its mRNA abundance with clinicopathological features (overall pathological stage, T, N, M categories and histological grade). Differences across ordered categories were tested with one‐way ANOVA (or Kruskal–Wallis when normality was not met). A nomogram incorporating DCAF7 and clinical variables was built with rms and calibrated at 1, 3 and 5 years; predictive accuracy was visualised with calibration plots. Diagnostic ability was quantified with ROC analysis using pROC and visualised in ggplot2. The AUC and its 95% confidence interval were reported, where an AUC of .5 indicates no discrimination and 1.0 denotes perfect diagnostic accuracy.

### 4.4 Genomic alteration analysis

The genomic profiles and mutational signatures of DCAF7 in different tumours were analysed using the cBioPortal platform (https://www.cbioportal.org).^53^ Putative copy number alternations (CNAs) were identified using the Genomic Identification of Significant Targets in Cancer to determine its relationship with DCAF7 mRNA expression. Mutation sites within DCAF7 were represented in the schematic diagram of its protein structure. The correlation between DCAF7 genetic alterations and LIHC prognosis was accessible within the ‘Comparison/Survival’ module. We also integrated related information on CNAs in pan‐cancer through the ‘mutation’ module in the Gene Set Cancer Analysis (GSCA) bioinformatic database (http://bioinfo.life.hust.edu.cn/GSCA).^54^ The LIHC patients were divided into two groups based on the median DCAF7 expression value. The somatic mutation landscape of these patients was visualised using SangerBox3.0, a bioinformation online tool (http://sangerbox.com/).

### 4.5 DNA methylation and mRNA modification analysis

DCAF7 promoter methylation in normal and pan‐cancer tissues were investigated using UALCAN. The level of DNA methylation is directly proportional to the beta value. DCAF7 DNA methylation status map at the CpG sites was obtained from the MethSurv database (https://biit.cs.ut.ee/methsurv/).^55^ Effects of methylation levels at these sites on the prognosis of LIHC patients was further evaluated. The correlation between DCAF7 expression and mRNA modification methylation regulatory factors in diverse cancer types was depicted by heatmaps with the ‘ggplot2’ package. The partial correlation coefficient (Cor) and its corresponding *p* value were generated by the Spearman's rank correlation test. Furthermore, the prediction of N(6)‐methyladenosine (m^6^A) modification locations on the DCAF7 RNA transcript was carried out utilising the SRAMP website (http://www.cuilab.cn/sramp/) without considering RNA secondary structure in liver tissue.^56^

### 4.6 Immune infiltration analysis

TIMER2.0 is a comprehensive resource for analysing the immunological characteristics of cancers in TCGA estimated by multiple immune deconvolution methods. The associations between DCAF7 expression and various immune cell types, such as B cells, CD4^+^ T cells, CD8^+^ T cells, endothelial cells, macrophages and NK cells, were determined by the EPIC algorithm and presented through a heatmap. Besides, scatterplots were generated to show the relationships of DCAF7 with particular immune infiltration levels or immune checkpoint expression levels in LIHC. Pan‐cancer data from TCGA and UCSC Xena databases were used to further profile the association coefficients between DCAF7 and 60 immune checkpoint genes. Based on the data from the TCGA‐LIHC project, enrichment scores for 24 immune cell types were quantified using the ssGSEA algorithm provided in the ‘GSVA’ R package.^57^, ^58^ We also employed the R package ‘ESTIMATE’ to compute the immune score, stromal score and estimate score for the individual LIHC sample.^59^ Single‐cell analysis of the DCAF7 expression status was conducted using the TISCH website (http://tisch.comp‐genomics.org/) and visually represented in UMAP plot.^60^ Furthermore, a heatmap was displayed to depict the expression levels of DCAF7 in distinct immune cell types, offering significant insights into its function within the TME. Utilising the CIBERSORT core algorithm, the difference in immune infiltration results between DCAF7 high and low expression groups was assessed, and a stacked bar graph was employed to illustrate the data distribution.^61^ The correlations between DCAF7 expression and MSI, TMB and NEO across various tumours from TCGA cohorts were obtained using the SangerBox platform. Spearman's correlation coefficients were calculated to generate the partial correlation and the *p* value. To minimise potential bias, all analyses were performed consistently across datasets, and results were cross‐checked for internal consistency. Limitations inherent to computational inference are acknowledged, and interpretations are made with appropriate caution.

### 4.7 DCAF7 interactome construction and drug sensitivity profiling

The top 100 genes exhibiting a similar expression pattern to DCAF7 in pan‐cancer were identified by the Gene Expression Profiling Interactive Analysis (GEPIA2) database (http://gepia2.cancer‐pku.cn).^62^ A network diagram of these genes was generated through the Cytoscape software. Hub genes were subsequently subjected to GO analysis and KEGG analysis with the ‘clusterProfiler’ R package to further investigate the potential function of DCAF7.^63^ Enriched GO terms and KEGG pathways were considered to be meaningful according to the criterion of adjusted *p* value < .05. The GDSC database (https://www.cancerrxgene.org/) is a public resource that provides information on drug sensitivity in cancer cells and molecular markers of drug response.^64^ The correlation between GDSC drug sensitivity and DCAF7 mRNA expression was analysed by GSCA. For hepatocellular carcinoma, the 15 genes most strongly correlated with DCAF7 were analysed pair‐wise; correlation matrices were displayed as heat maps (ggplot2) and chord diagrams (circlise).^65^ The prospective pharmaceutical compounds were predicted from the Drug Signatures Database (DSigDB) retrieved via the Enrichr platform (http://amp.pharm.mssm.edu/Enrichr) based on these genes.^66^ We also utilised the NetworkAnalyst database (https://www.networkanalyst.ca/)^67^ and the CTD (https://ctdbase.org/)^68^ to construct a protein–chemical interaction network. The minimum network was then imported into Cytoscape software to generate a network diagram.

### 4.8 Identification of DEGs and functional enrichment analysis

CancerSEA is a database capable of portraying the functional status atlas of cancer cells at the single‐cell level.^69^ We used CancerSEA to explore the average correlation between DCAF7 and 14 cancer functional states. The threshold was set at a correlation strength of .3 and a *p* value of less than .05. Based on the median expression level of DCAF7 in TCGA‐LIHC public data, samples were classified into cohorts exhibiting high and low DCAF7 expression. The ‘DESeq2’ R package was employed to perform differential gene expression analysis, with the |log2FoldChange (logFC)| > 1 and adjusted *p* value < .05 as the criteria for DEGs identification.^70^ The top 5 up‐regulated and down‐regulated DEGs related to DCAF7 were selected for Spearman correlation analysis and the results were presented as a heatmap via the ‘ggplot2’. Furthermore, GO, KEGG and GSEA were conducted on DEGs using the ‘clusterProfiler’ R package. For GSEA analysis, the c2.cp.all.v2022.1.Hs.symbols.gmt curated gene sets were collected from the Molecular Signatures Database.^71^ Significance was established when the adjusted *p* value < .05 and the false discovery rate < .25. A mountain plot was generated using ‘ggplot2’ for result visualisation.

### 4.9 Cell lines and culture conditions

The human hepatoma cell lines HepG2 and Huh7 were obtained from the Chinese Academy of Science (Shanghai, China). HepG2 was cultured in MEM (Gibco, USA), and Huh7 was cultured in DMEM medium (Gibco) with 10% foetal bovine serum (FBS) (Gibco) and 100 U/mL penicillin–streptomycin (P/S). Isolated CD8^+^ T cells were cultured in RPMI medium (Gibco) containing 10% FBS and 100 U/mL P/S supplemented with 100 IU/mL rhIL2 (Beyotime, Shanghai, China; #P4777). All cells were incubated in an incubator containing 5% CO_2_ at 37°C.

### 4.10 Plasmids and CRISPR–Cas9 system

The pLV–DCAF7–Myc lentiviral vectors and corresponding control vectors were used for gain‐of‐function studies. All lentiviral vectors were constructed by Shangya Biotechnology Co., Ltd. (Fuzhou, China). All transductions were performed according to the manufacturer's instructions. For CRISPR–Cas9‐mediated knockout of DCAF7, HepG2 and Huh7 cells were transfected using the TurboCRISPR KO Kit (#DZH301) from Qingke Biotechnology Co., Ltd (Beijing, China) according to the manufacturer's protocol. Two independent sgRNAs targeting DCAF7 were designed and validated for knockout efficiency, including sg#1 with the sequence 5′‐ACGCGATGAACTGGAGTGTG‐3′ and sg#2 with the sequence 5′‐GTCCCTGCACGGCAAACGGA‐3′. Cas9 nuclease and sgRNA complexes were delivered via specially treated carriers to enhance transfection efficiency, and successful knockout was confirmed by immunoblot analysis.

### 4.11 RNA isolation and quantitative real‐time PCR

Total RNA from LIHC cells was isolated using the RNA Isolater Total RNA Extraction Reagent (Vazyme, Nanjing, China), and cDNA was synthesised using the Reverse Transcription Kit (Vazyme) according to the manufacturer's instructions. mRNA expression was detected using the SYBR Green PCR Kit (Vazyme). The primer sequences are listed in Table S19.

For RNA stability analysis, HepG2 cells were treated with actinomycin D (5 µg/mL; Sigma) to inhibit transcription and collected at the indicated time points. RNA was extracted and reverse‐transcribed, and DCAF7 mRNA stability was evaluated by qPCR based on the relative residual transcript levels.

### 4.12 Western blot and Co‐IP

Cell lysates were prepared using ice‐cold RIPA buffer supplemented with the protease inhibitor. Protein samples were separated through SDS‐PAGE gels and electro‐transferred to PVDF membranes (Merck Millipore, USA). After blocking with 5% nonfat milk, membranes were incubated overnight at 4°C with the following primary antibodies: DCAF7 (Abcam; #ab138490, 1:1000), Myc‐Tag (ABclonal; #AE070, 1:3000), YTHDC1 (CST; #77422, 1:1000), p‐GSK‐3β (CST; #5558, 1:1000), GSK‐3β (CST; #12456, 1:1000; CST; #9832, 1:1000), β‐catenin (CST; #8480, 1:1000), c‐Myc (Proteintech; #10828‐1‐AP, 1:1000) and cyclin D1 (ZENBIO; #R380999, 1:1000). GAPDH antibody (Proteintech; #60004‐1‐Ig) served as loading control at 1:5000 dilution. Protein bands were visualised by ECL solution after appropriate secondary antibody incubation.

For immunoprecipitation, total protein lysates were incubated with Protein A/G PLUS‐Agarose beads (Santa Cruz; #sc‐2003) and the indicated antibodies under gentle rotation overnight at 4°C. After three washes, the immunocomplexes were analysed by immunoblotting.

### 4.13 RNA immunoprecipitation

RIP assays were performed using the RIP Kit (Bersin Biotechnology Co., Ltd., Guangzhou, China; #Bes5101) following the manufacturer's instructions. Briefly, HepG2 cells were washed three times with ice‐cold PBS and lysed in RIP lysis buffer. The cell lysates were incubated with either control IgG or anti‐YTHDC1 antibodies at 4°C to allow formation of RNA–protein complexes, followed by the addition of magnetic beads to capture the immune complexes. After washing to remove unbound material, the immunoprecipitated RNA was purified and subjected to qPCR analysis to detect target RNA enrichment.

### 4.14 Methylated RIP

MeRIP assays were conducted using the m^6^A MeRIP Kit (Bersin Biotechnology Co., Ltd.; #Bes5203‐2) according to the manufacturer's protocol. Briefly, total RNA was extracted with TRIzol reagent and sheared into approximately 300 nt fragments using the RNA fragmentation buffer provided in the kit. The resulting RNA fragments were incubated with either anti‐m^6^A antibodies or control IgG at 4°C for 4 h, followed by the addition of Protein A/G magnetic beads for an additional 2 h to capture the immune complexes. After extensive washing to remove unbound RNA, the bead‐bound RNA was extracted, purified and reverse‐transcribed into cDNA. The enrichment of DCAF7 mRNA in the immunoprecipitated fraction was quantified by qPCR. The primer sequences are listed in Table S19.

### 4.15 HepG2–T cell co‐culture assay

Peripheral blood mononuclear cells (PBMCs) were purchased from Milecell Biotechnologies (Shanghai, China, #PB025C/PB025C‐W). T cells were then purified from PBMCs using the EasySep™ Human CD8^+^ T Cell Isolation Kit (STEMCELL Technologies, Vancouver, Canada; #17953) according to the manufacturer's instructions. Isolated CD8^+^ T cells were activated with the ImmunoCult™ Human CD3/CD28 T Cell Activator (STEMCELL; #10971) for 72 h, then co‐cultured with DCAF7 knockout or control HepG2 cells at an effector‐to‐target (E:T) ratio of 3:1 or 5:1. After 48 h, the supernatants were harvested for ELISA analysis.

### 4.16 Enzyme‐linked immunosorbent assay

Cell culture supernatants were harvested and clarified by centrifugation at 1000×*g* for 20 min. The concentration of human IFN‐γ was determined using a Human IFN‐γ High Sensitivity ELISA Kit (Lianke Biotech, Hangzhou, China; #EK180HS) according to the manufacturer's instructions. Absorbance was recorded at 450 nm using a microplate reader.

### 4.17 Cell viability assay

Cells infected with DCAF7 overexpression or knockdown lentivirus were plated in 96‐well plates (3 × 10^3^ cells per well) in triplicate and maintained for indicated days. Cellular proliferation was assessed using MTS reagent (Promega; G3580) following the manufacturer's protocol, with metabolic activity quantified through 490 nm absorbance measurements.

### 4.18 Colony formation assay

Transfected cells were seeded in six‐well plates (500 cells per well) and cultured for 14 days to permit colony formation. Developed colonies were fixed with 4% paraformaldehyde solution for 15 min followed by crystal violet staining. Colony quantification was performed through densitometric analysis using image J.

### 4.19 Wound‐scratch assay

Confluent cell monolayers in culture dishes were linearly wounded using 200 µL pipette tips. Initial wound images (0 h) were captured immediately post‐scratching using phase‐contrast microscopy. After indicated incubation under serum‐free culture conditions, corresponding wound areas were reacquired. Quantitative analysis was performed with Image J software by comparing the residual wound area to the initial measurement. The migratory closure rate was quantified: closure (%) = [(*A*_0_ – *A*_24_)/*A*_0_] × 100, where *A*_0_ represents the initial wound area and *A*_24_ represents the remaining area after 24 h.

### 4.20 Transwell assay

Cell migration assay was performed using transwell chambers assembled in 24‐well plates. DCAF7‐knockdown and DCAF7‐overexpression LIHC cells suspended with serum‐free medium were loaded into the upper chamber, and 600 µL complete medium (10% FBS) was added to the lower chamber. After incubation for 48 h, non‐migrated cells were removed by a cotton swab. Migrated cells were fixed with 4% paraformaldehyde for 30 min and stained with .1% crystal violet. Following three PBS washes, membranes were air‐dried and visualised under an inverted microscope for quantitative analysis.

### 4.21 Statistical analysis

Statistical analyses in this research were performed utilising the aforementioned online database and the R packages. GraphPad Prism 9.0 software was used for the statistical analysis of the in vitro experimental data. Two‐tailed Student's *t*‐test was performed to calculate the *p* value of two subgroups and data in bar graphs represent mean ± SD of a minimum of three biological replicates. Statistical significance was indicated at *p < .05, **p < .01 and ***p < .001.

## AUTHOR CONTRIBUTIONS

**Ruina Luan**: Writing‐original draft, visualization, validation, software, methodology, investigation, formal analysis, data curation, conceptualization. **Hanbin Lin**: Writing‐original draft, visualization, validation, methodology, investigation, formal analysis, data curation. **Xin Zhao**: Writing‐original draft, visualization, validation, methodology, investigation, formal analysis, data curation. **Jianpeng Li**: Software, methodology, data curation, visualization. **Maohe Chen**: Writing‐review & editing, resources, validation, project administration, formal analysis, data curation, conceptualization. **Shiping Luo**: Writing‐review & editing, resources, validation, project administration, formal analysis, data curation, conceptualization. **Xinjian Lin**: Writing‐original draft, writing‐review & editing, resources, validation, project administration, funding acquisition, formal analysis, data curation, conceptualization. All authors read and approved the final manuscript.

## CONFLICT OF INTEREST STATEMENT

The authors declare no conflicts of interest.

## Supporting information



Supporting information

Supporting information

## Data Availability

All datasets analysed during this study are included in the article or in the supporting information. Further inquiries can be directed to the corresponding author.

## References

[1] Bray F , Laversanne M , Sung H , et al. Global cancer statistics 2022: GLOBOCAN estimates of incidence and mortality worldwide for 36 cancers in 185 countries. CA Cancer J Clin. 2024;74(3):229‐263. doi:10.3322/caac.21834 38572751

[2] Bray F , Laversanne M , Weiderpass E , Soerjomataram I . The ever‐increasing importance of cancer as a leading cause of premature death worldwide. Cancer. 2021;127(16):3029‐3030. doi:10.1002/cncr.33587 34086348

[3] Chen S , Cao Z , Prettner K , et al. Estimates and projections of the global economic cost of 29 cancers in 204 countries and territories from 2020 to 2050. JAMA Oncol. 2023;9(4):465‐472. doi:10.1001/jamaoncol.2022.7826 36821107 PMC9951101

[4] Srivastava S , Hanash S . Pan‐cancer early detection: hype or hope?. Cancer Cell. 2020;38(1):23‐24. doi:10.1016/j.ccell.2020.05.021 32531269

[5] Vis DJ , Jaaks P , Aben N , et al. A pan‐cancer screen identifies drug combination benefit in cancer cell lines at the individual and population level. Cell Rep Med. 2024;5(8):101687. doi:10.1016/j.xcrm.2024.101687 39168097 PMC11384948

[6] Wegmann R , Bankel L , Festl Y , et al. Molecular and functional landscape of malignant serous effusions for precision oncology. Nat Commun. 2024;15(1):8544. doi:10.1038/s41467-024-52694-8 39358333 PMC11447229

[7] DiRusso CJ , Dashtiahangar M , Gilmore TD . Scaffold proteins as dynamic integrators of biological processes. J Biol Chem. 2022;298(12):102628. doi:10.1016/j.jbc.2022.102628 36273588 PMC9672449

[8] Faux MC , Scott JD . Molecular glue: kinase anchoring and scaffold proteins. Cell. 1996;85(1):9‐12. doi:10.1016/s0092-8674(00)81075-2 8620541

[9] Shaw AS , Filbert EL . Scaffold proteins and immune‐cell signalling. Nat Rev Immunol. 2009;9(1):47‐56. doi:10.1038/nri2473 19104498

[10] Stirnimann CU , Petsalaki E , Russell RB , Müller CW . WD40 proteins propel cellular networks. Trends Biochem Sci. 2010;35(10):565‐574. doi:10.1016/j.tibs.2010.04.003 20451393

[11] Peng Z , Liao Z , Matsumoto Y , Yang A , Tomkinson AE . Human DNA ligase I interacts with and is targeted for degradation by the DCAF7 specificity factor of the Cul4‐DDB1 ubiquitin ligase complex. J Biol Chem. 2016;291(42):21893‐21902. doi:10.1074/jbc.M116.746198 27573245 PMC5063974

[12] Xu J , Ye Z , Zhuo Q , et al. MEN1 degradation induced by neddylation and the CUL4B‐DCAF7 axis promotes pancreatic neuroendocrine tumor progression. Cancer Res. 2023;83(13):2226‐2247. doi:10.1158/0008-5472.Can-22-3599 36939378

[13] Li QJ , Fang XL , Li YQ , et al. DCAF7 acts as a scaffold to recruit USP10 for G3BP1 deubiquitylation and facilitates chemoresistance and metastasis in nasopharyngeal carcinoma. Adv Sci (Weinh). 2024;11(36):e2403262. doi:10.1002/advs.202403262 38973296 PMC11423104

[14] Glenewinkel F , Cohen MJ , King CR , et al. The adaptor protein DCAF7 mediates the interaction of the adenovirus E1A oncoprotein with the protein kinases DYRK1A and HIPK2. Sci Rep. 2016;6:28241. doi:10.1038/srep28241 27307198 PMC4910162

[15] Kiri S , Ryba T . Cancer, metastasis, and the epigenome. Mol Cancer. 2024;23(1):154. doi:10.1186/s12943-024-02069-w 39095874 PMC11295362

[16] Smith ZD , Hetzel S , Meissner A . DNA methylation in mammalian development and disease. Nat Rev Genet. 2025;26(1):7‐30. doi:10.1038/s41576-024-00760-8 39134824

[17] Lin S , Kuang M . RNA modification‐mediated mRNA translation regulation in liver cancer: mechanisms and clinical perspectives. Nat Rev Gastroenterol Hepatol. 2024;21(4):267‐281. doi:10.1038/s41575-023-00884-y 38243019

[18] Zhao BS , Roundtree IA , He C . Post‐transcriptional gene regulation by mRNA modifications. Nat Rev Mol Cell Biol. 2017;18(1):31‐42. doi:10.1038/nrm.2016.132 27808276 PMC5167638

[19] Flamand MN , Tegowski M , Meyer KD . The proteins of mRNA modification: writers, readers, and erasers. Annu Rev Biochem. 2023;92:145‐173. doi:10.1146/annurev-biochem-052521-035330 37068770 PMC10443600

[20] Elhanani O , Ben‐Uri R , Keren L . Spatial profiling technologies illuminate the tumor microenvironment. Cancer Cell. 2023;41(3):404‐420. doi:10.1016/j.ccell.2023.01.010 36800999

[21] Kureshi CT , Dougan SK . Cytokines in cancer. Cancer Cell. 2025;43(1):15‐35. doi:10.1016/j.ccell.2024.11.011 39672170 PMC11841838

[22] Liu Y , Wang Y , Yang Y , et al. Emerging phagocytosis checkpoints in cancer immunotherapy. Signal Transduct Target Ther. 2023;8(1):104. doi:10.1038/s41392-023-01365-z 36882399 PMC9990587

[23] Xie N , Shen G , Gao W , Huang Z , Huang C , Fu L . Neoantigens: promising targets for cancer therapy. Signal Transduct Target Ther. 2023;8(1):9. doi:10.1038/s41392-022-01270-x 36604431 PMC9816309

[24] Wang X , Lamberti G , Di Federico A , et al. Tumor mutational burden for the prediction of PD‐(L)1 blockade efficacy in cancer: challenges and opportunities. Ann Oncol. 2024;35(6):508‐522. doi:10.1016/j.annonc.2024.03.007 38537779

[25] Zhao P , Li L , Jiang X , Li Q . Mismatch repair deficiency/microsatellite instability‐high as a predictor for anti‐PD‐1/PD‐L1 immunotherapy efficacy. J Hematol Oncol. 2019;12(1):54. doi:10.1186/s13045-019-0738-1 31151482 PMC6544911

[26] Frame S , Cohen P , Biondi RM . A common phosphate binding site explains the unique substrate specificity of GSK3 and its inactivation by phosphorylation. Mol Cell. 2001;7(6):1321‐1327. doi:10.1016/s1097-2765(01)00253-2 11430833

[27] de Vetten N , Quattrocchio F , Mol J , Koes R . The an11 locus controlling flower pigmentation in petunia encodes a novel WD‐repeat protein conserved in yeast, plants, and animals. Genes Dev. 1997;11(11):1422‐1434. doi:10.1101/gad.11.11.1422 9192870

[28] Nissen RM , Amsterdam A , Hopkins N . A zebrafish screen for craniofacial mutants identifies wdr68 as a highly conserved gene required for endothelin‐1 expression. BMC Dev Biol. 2006;6:28. doi:10.1186/1471-213x-6-28 16759393 PMC1523201

[29] Morriss GR , Jaramillo CT , Mikolajczak CM , Duong S , Jaramillo MS , Cripps RM . The Drosophila wings apart gene anchors a novel, evolutionarily conserved pathway of neuromuscular development. Genetics. 2013;195(3):927‐940. doi:10.1534/genetics.113.154211 24026097 PMC3813874

[30] Morita K , Lo Celso C , Spencer‐Dene B , Zouboulis CC , Watt FM . HAN11 binds mDia1 and controls GLI1 transcriptional activity. J Dermatol Sci. 2006;44(1):11‐20. doi:10.1016/j.jdermsci.2006.06.001 16887337

[31] Ritterhoff S , Farah CM , Grabitzki J , Lochnit G , Skurat AV , Schmitz ML . The WD40‐repeat protein Han11 functions as a scaffold protein to control HIPK2 and MEKK1 kinase functions. EMBO J. 2010;29(22):3750‐3761. doi:10.1038/emboj.2010.251 20940704 PMC2989105

[32] Yu D , Cattoglio C , Xue Y , Zhou Q . A complex between DYRK1A and DCAF7 phosphorylates the C‐terminal domain of RNA polymerase II to promote myogenesis. Nucleic Acids Res. 2019;47(9):4462‐4475. doi:10.1093/nar/gkz162 30864669 PMC6511856

[33] Frendo‐Cumbo S , Li T , Ammendolia DA , et al. DCAF7 regulates cell proliferation through IRS1‐FOXO1 signaling. iScience. 2022;25(10):105188. doi:10.1016/j.isci.2022.105188 36248734 PMC9556925

[34] Qin Q , Ruan H , Zhang H , et al. Deubiquitinase MYSM1: an important tissue development and function regulator. Int J Mol Sci. 2024;25(23). doi:10.3390/ijms252313051 PMC11641604 39684760

[35] Ju Y , Fang S , Liu L , Ma H , Zheng L . The function of the ELF3 gene and its mechanism in cancers. Life Sci. 2024;346:122637. doi:10.1016/j.lfs.2024.122637 38614305

[36] Li Y , Porta‐Pardo E , Tokheim C , et al. Pan‐cancer proteogenomics connects oncogenic drivers to functional states. Cell. 2023;186(18):3921‐3944. doi:10.1016/j.cell.2023.07.014. e25. 37582357

[37] Martincorena I , Campbell PJ . Somatic mutation in cancer and normal cells. Science. 2015;349(6255):1483‐1489. doi:10.1126/science.aab4082 26404825

[38] Dawson MA , Kouzarides T . Cancer epigenetics: from mechanism to therapy. Cell. 2012;150(1):12‐27. doi:10.1016/j.cell.2012.06.013 22770212

[39] Nishiyama A , Nakanishi M . Navigating the DNA methylation landscape of cancer. Trends Genet. 2021;37(11):1012‐1027. doi:10.1016/j.tig.2021.05.002 34120771

[40] Hinshaw DC , Shevde LA . The tumor microenvironment innately modulates cancer progression. Cancer Res. 2019;79(18):4557‐4566. doi:10.1158/0008-5472.Can-18-3962 31350295 PMC6744958

[41] Glaviano A , Lau HS , Carter LM , et al. Harnessing the tumor microenvironment: targeted cancer therapies through modulation of epithelial‐mesenchymal transition. J Hematol Oncol. 2025;18(1):6. doi:10.1186/s13045-024-01634-6 39806516 PMC11733683

[42] He X , Xu C . Immune checkpoint signaling and cancer immunotherapy. Cell Res. 2020;30(8):660‐669. doi:10.1038/s41422-020-0343-4 32467592 PMC7395714

[43] Angers S , Moon RT . Proximal events in Wnt signal transduction. Nat Rev Mol Cell Biol. 2009;10(7):468‐477. doi:10.1038/nrm2717 19536106

[44] Ruiz de Galarreta M , Bresnahan E , Molina‐Sánchez P , et al. β‐Catenin activation promotes immune escape and resistance to anti‐PD‐1 therapy in hepatocellular carcinoma. Cancer Discov. 2019;9(8):1124‐1141. doi:10.1158/2159-8290.Cd-19-0074 31186238 PMC6677618

[45] Xu C , Xu Z , Zhang Y , Evert M , Calvisi DF , Chen X . Catenin signaling in hepatocellular carcinoma. J Clin Invest. 2022;132(4). doi:10.1172/jci154515 PMC8843739 35166233

[46] Mariotti L , Pollock K , Guettler S . Regulation of Wnt/β‐catenin signalling by tankyrase‐dependent poly(ADP‐ribosyl)ation and scaffolding. Br J Pharmacol. 2017;174(24):4611‐4636. doi:10.1111/bph.14038 28910490 PMC5727255

[47] Goldman MJ , Craft B , Hastie M , et al. Visualizing and interpreting cancer genomics data via the Xena platform. Nat Biotechnol. 2020;38(6):675‐678. doi:10.1038/s41587-020-0546-8 32444850 PMC7386072

[48] Vivian J , Rao AA , Nothaft FA , et al. Toil enables reproducible, open source, big biomedical data analyses. Nat Biotechnol. 2017;35(4):314‐316. doi:10.1038/nbt.3772 28398314 PMC5546205

[49] Li T , Fu J , Zeng Z , et al. TIMER2.0 for analysis of tumor‐infiltrating immune cells. Nucleic Acids Res. 2020;48(W1):W509‐w514. doi:10.1093/nar/gkaa407 32442275 PMC7319575

[50] Chandrashekar DS , Karthikeyan SK , Korla PK , et al. UALCAN: an update to the integrated cancer data analysis platform. Neoplasia. 2022;25:18‐27. doi:10.1016/j.neo.2022.01.001 35078134 PMC8788199

[51] Thul PJ , Lindskog C . The human protein atlas: a spatial map of the human proteome. Protein Sci. 2018;27(1):233‐244. doi:10.1002/pro.3307 28940711 PMC5734309

[52] Liu J , Lichtenberg T , Hoadley KA , et al. An integrated TCGA pan‐cancer clinical data resource to drive high‐quality survival outcome analytics. Cell. 2018;173(2):400‐416. doi:10.1016/j.cell.2018.02.052. e11. 29625055 PMC6066282

[53] Cerami E , Gao J , Dogrusoz U , et al. The cBio cancer genomics portal: an open platform for exploring multidimensional cancer genomics data. Cancer Discov. 2012;2(5):401‐404. doi:10.1158/2159-8290.Cd-12-0095 22588877 PMC3956037

[54] Liu CJ , Hu FF , Xie GY , et al. GSCA: an integrated platform for gene set cancer analysis at genomic, pharmacogenomic and immunogenomic levels. Brief Bioinform. 2023;24(1). doi:10.1093/bib/bbac558 36549921

[55] Modhukur V , Iljasenko T , Metsalu T , Lokk K , Laisk‐Podar T , Vilo J . MethSurv: a web tool to perform multivariable survival analysis using DNA methylation data. Epigenomics. 2018;10(3):277‐288. doi:10.2217/epi-2017-0118 29264942

[56] Zhou Y , Zeng P , Li YH , Zhang Z , Cui Q . SRAMP: prediction of mammalian N6‐methyladenosine (m6A) sites based on sequence‐derived features. Nucleic Acids Res. 2016;44(10):e91. doi:10.1093/nar/gkw104 26896799 PMC4889921

[57] Bindea G , Mlecnik B , Tosolini M , et al. Spatiotemporal dynamics of intratumoral immune cells reveal the immune landscape in human cancer. Immunity. 2013;39(4):782‐795. doi:10.1016/j.immuni.2013.10.003 24138885

[58] Hänzelmann S , Castelo R , Guinney J . GSVA: gene set variation analysis for microarray and RNA‐seq data. BMC Bioinformatics. 2013;14. doi:10.1186/1471-2105-14-7 PMC3618321 23323831

[59] Yoshihara K , Shahmoradgoli M , Martínez E , et al. Inferring tumour purity and stromal and immune cell admixture from expression data. Nat Commun. 2013;4:2612. doi:10.1038/ncomms3612 24113773 PMC3826632

[60] Sun D , Wang J , Han Y , et al. TISCH: a comprehensive web resource enabling interactive single‐cell transcriptome visualization of tumor microenvironment. Nucleic Acids Res. 2021;49(D1):D1420‐d1430. doi:10.1093/nar/gkaa1020 33179754 PMC7778907

[61] Newman AM , Liu CL , Green MR , et al. Robust enumeration of cell subsets from tissue expression profiles. Nat Methods. 2015;12(5):453‐457. doi:10.1038/nmeth.3337 25822800 PMC4739640

[62] Tang Z , Kang B , Li C , Chen T , Zhang Z . GEPIA2: an enhanced web server for large‐scale expression profiling and interactive analysis. Nucleic Acids Res. 2019;47(W1):W556‐w560. doi:10.1093/nar/gkz430 31114875 PMC6602440

[63] Yu G , Wang LG , Han Y , He QY . clusterProfiler: an R package for comparing biological themes among gene clusters. OMICS. 2012;16(5):284‐287. doi:10.1089/omi.2011.0118 22455463 PMC3339379

[64] Yang W , Soares J , Greninger P , et al. Genomics of drug sensitivity in cancer (GDSC): a resource for therapeutic biomarker discovery in cancer cells. Nucleic Acids Res. 2013;41(Database issue):D955‐61. doi:10.1093/nar/gks1111 23180760 PMC3531057

[65] Gu Z , Gu L , Eils R , Schlesner M , Brors B . circlize Implements and enhances circular visualization in R. Bioinformatics. 2014;30(19):2811‐2812. doi:10.1093/bioinformatics/btu393 24930139

[66] Kuleshov MV , Jones MR , Rouillard AD , et al. Enrichr: a comprehensive gene set enrichment analysis web server 2016 update. Nucleic Acids Res. 2016;44(W1):W90‐97. doi:10.1093/nar/gkw377 27141961 PMC4987924

[67] Zhou G , Soufan O , Ewald J , Hancock REW , Basu N , Xia J . NetworkAnalyst 3.0: a visual analytics platform for comprehensive gene expression profiling and meta‐analysis. Nucleic Acids Res. 2019;47(W1):W234‐w241. doi:10.1093/nar/gkz240 30931480 PMC6602507

[68] Davis AP , Wiegers TC , Johnson RJ , Sciaky D , Wiegers J , Mattingly CJ . Comparative toxicogenomics database (CTD): update 2023. Nucleic Acids Res. 2023;51(D1):D1257‐d1262. doi:10.1093/nar/gkac833 36169237 PMC9825590

[69] Yuan H , Yan M , Zhang G , et al. CancerSEA: a cancer single‐cell state atlas. Nucleic Acids Res. 2019;47(D1):D900‐d908. doi:10.1093/nar/gky939 30329142 PMC6324047

[70] Love MI , Huber W , Anders S . Moderated estimation of fold change and dispersion for RNA‐seq data with DESeq2. Genome Biol. 2014;15(12):550. doi:10.1186/s13059-014-0550-8 25516281 PMC4302049

[71] Liberzon A , Birger C , Thorvaldsdóttir H , Ghandi M , Mesirov JP , Tamayo P . The molecular signatures database (MSigDB) hallmark gene set collection. Cell Syst. 2015;1(6):417‐425. doi:10.1016/j.cels.2015.12.004 26771021 PMC4707969

