# Supplementary figures and images for: Pan‐cancer multi‐omics reveals DCAF7 as an immune‐modulating prognostic driver and Wnt/β‐catenin activator in hepatocellular carcinoma
Source: Clin Transl Med. 2025 Dec 31;16(1):e70572. doi: 10.1002/ctm2.70572 (PMC12754573; doi:10.1002/ctm2.70572)

Figure S1

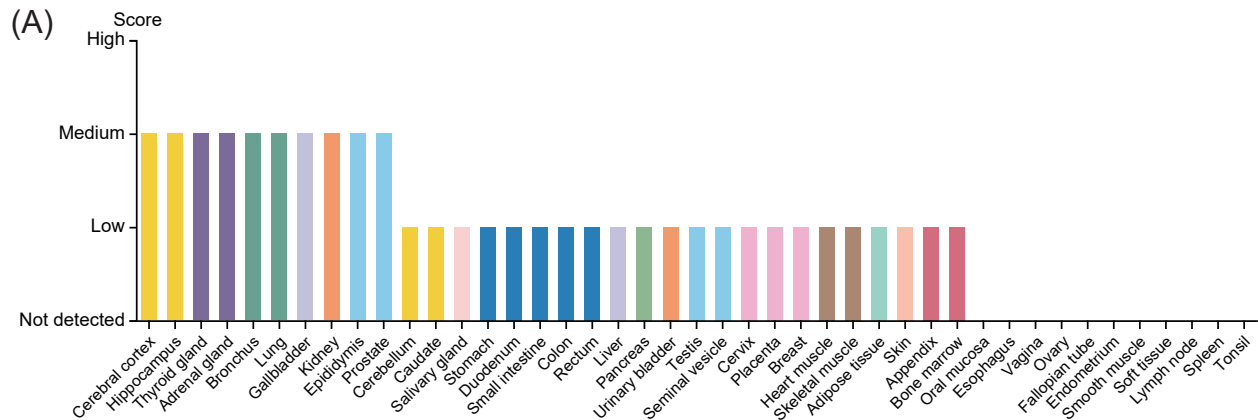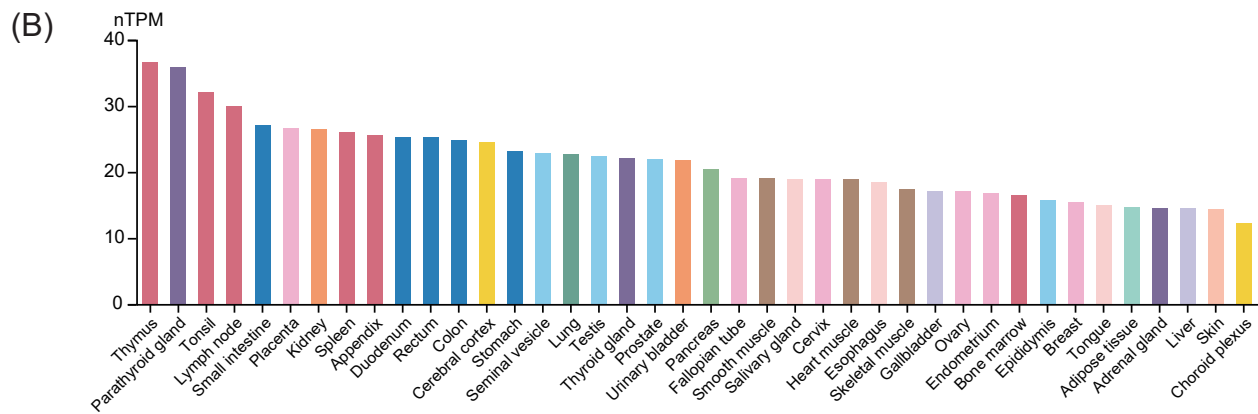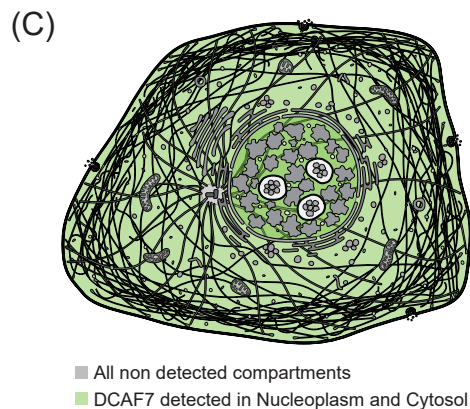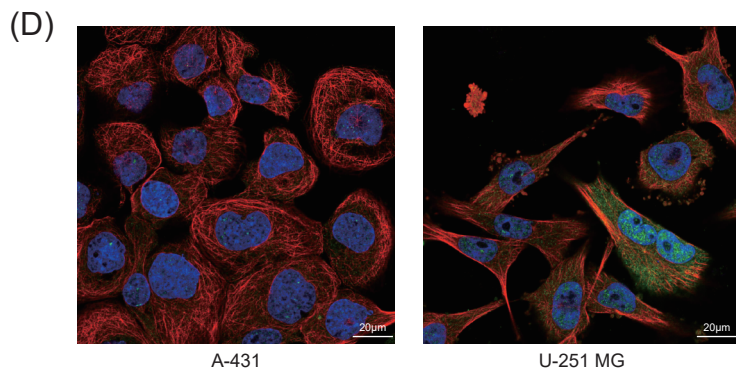

Figure S2

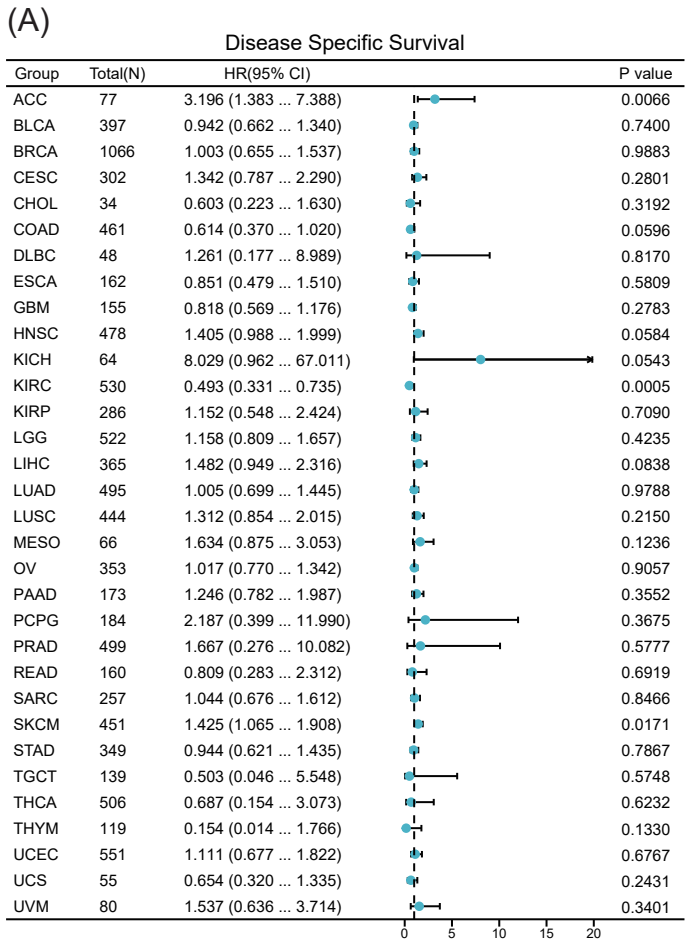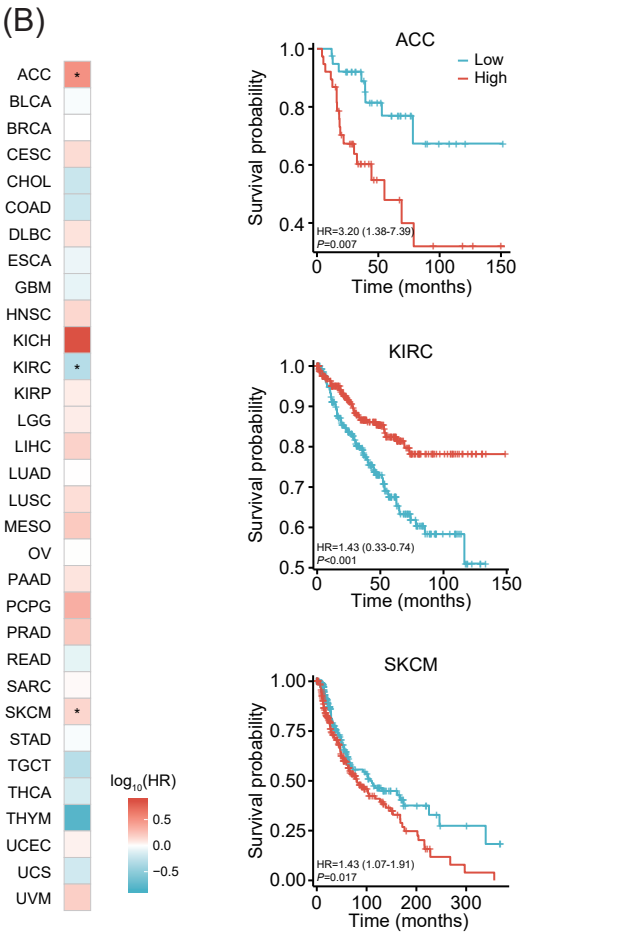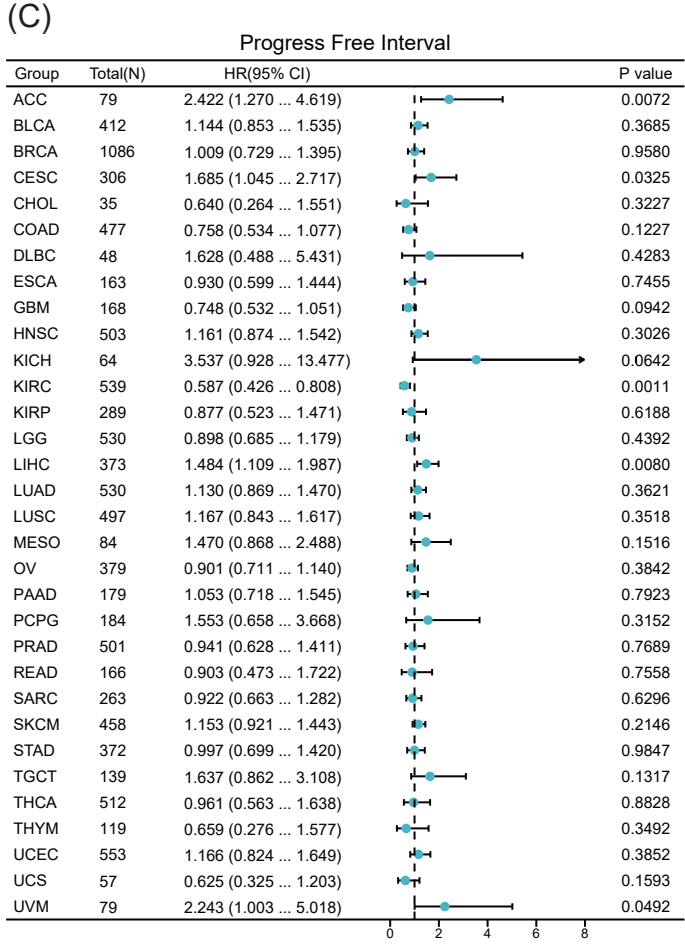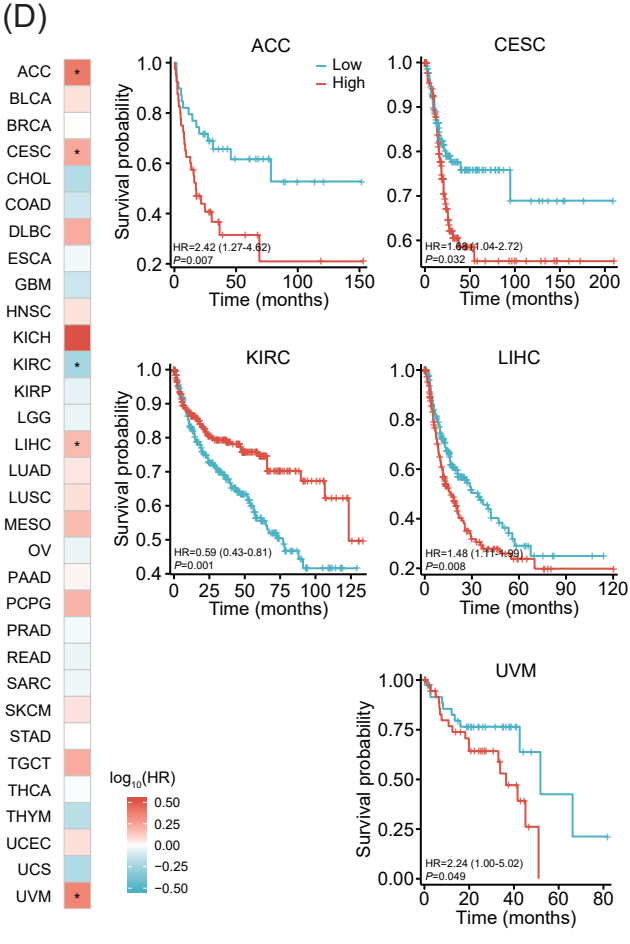

Figure S3

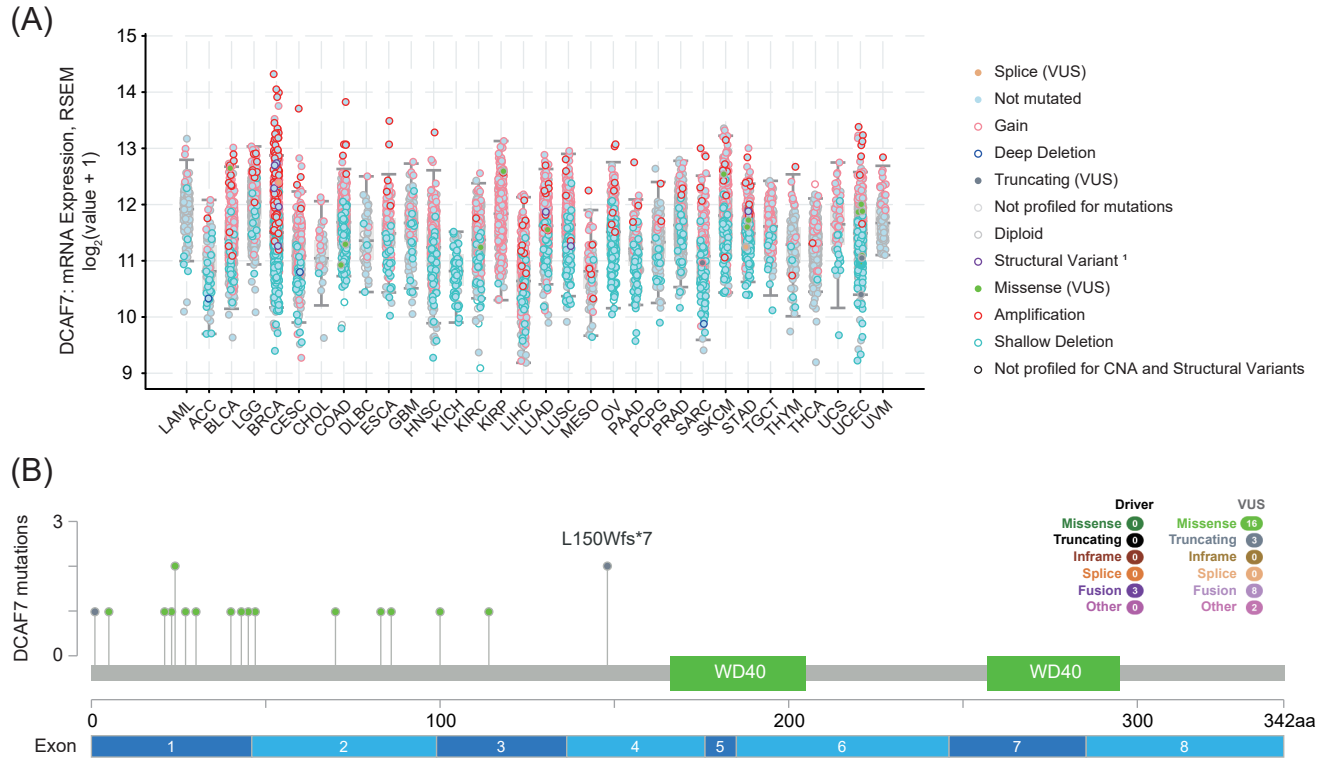

# Figure S4

(A)

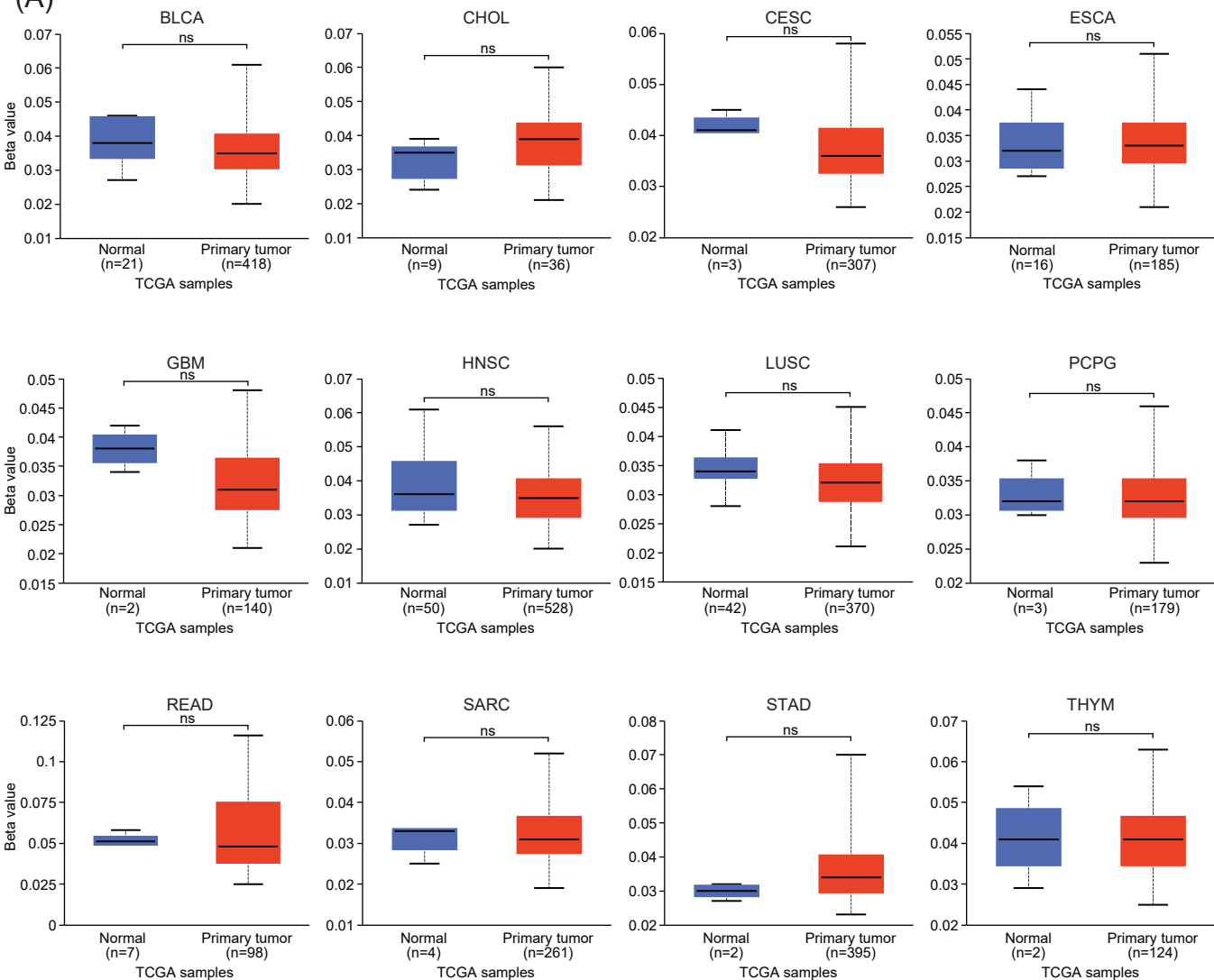

Figure S5

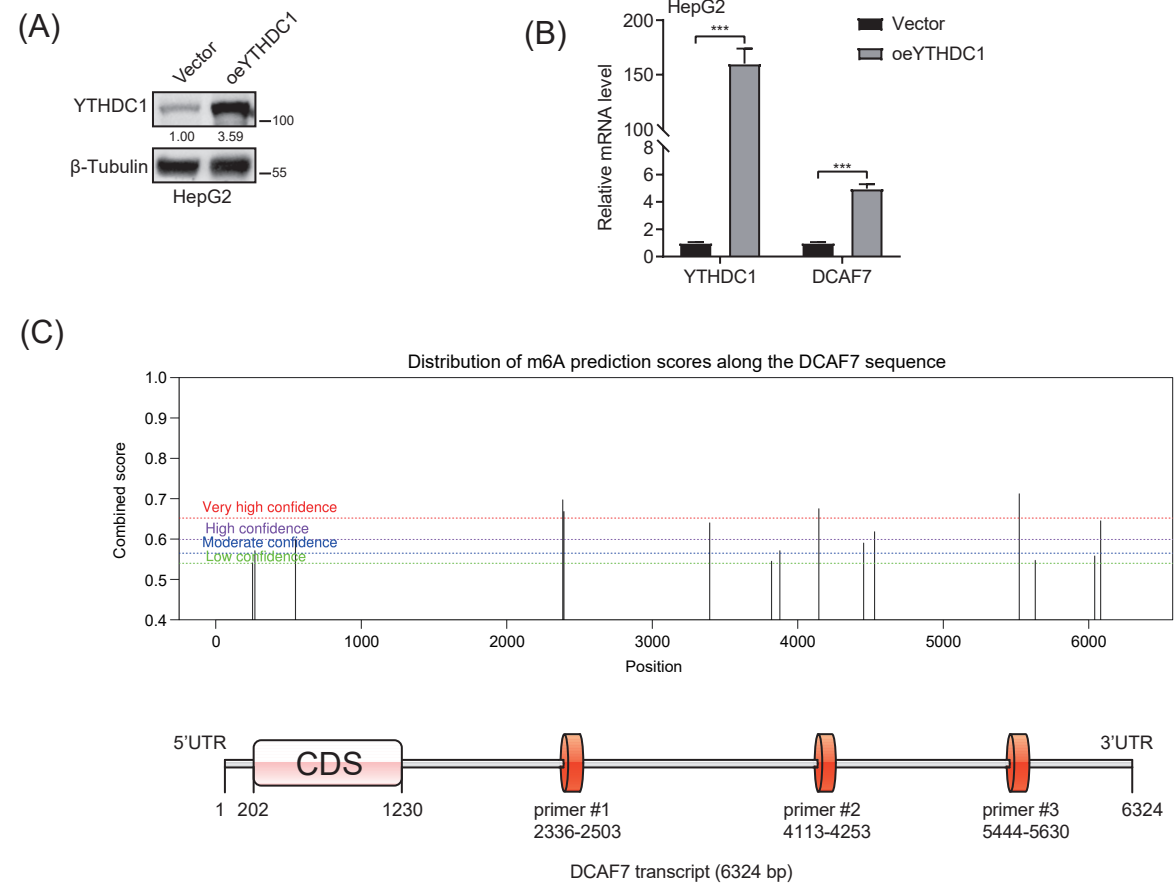

Figure S6

(A)

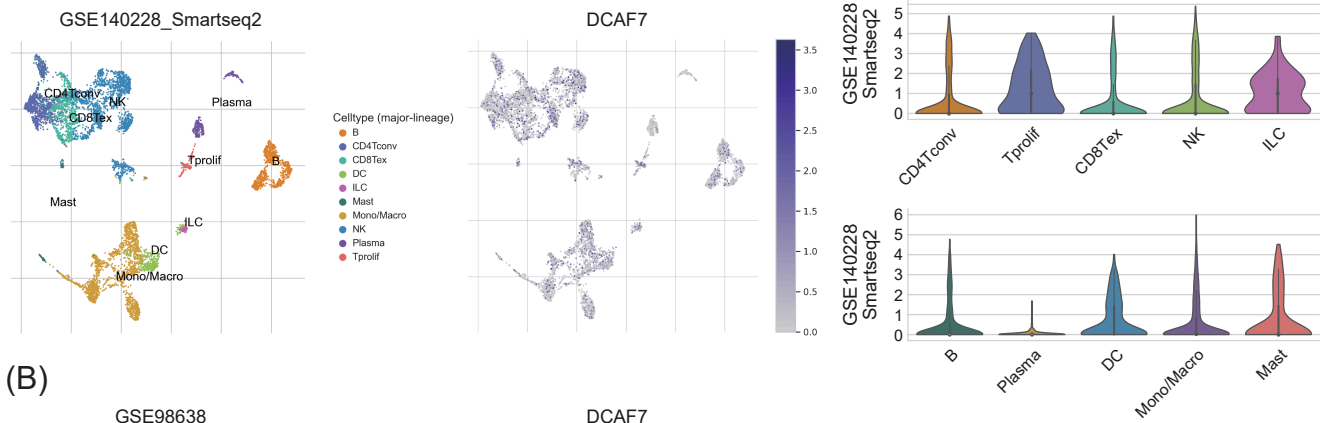

(B)

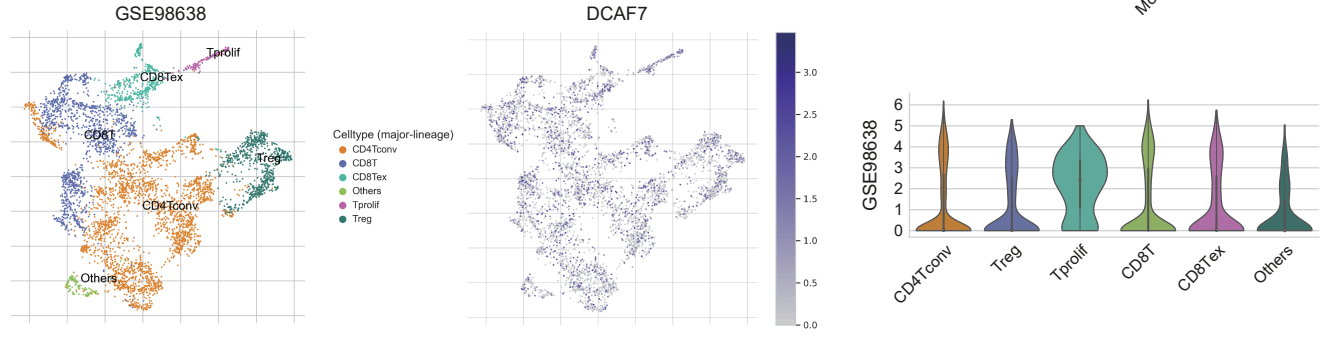

(C)

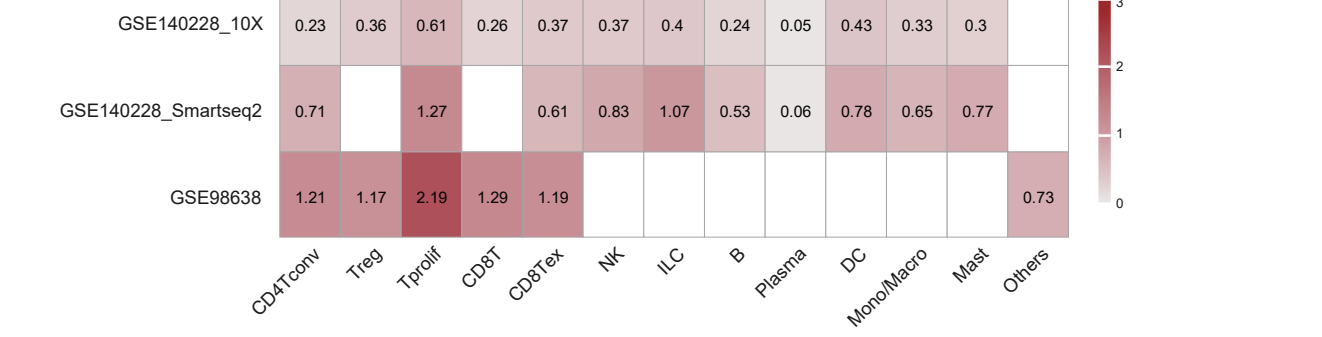

(D)

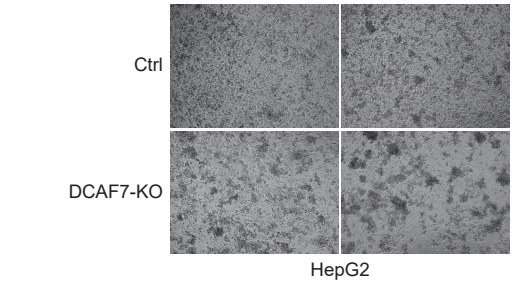

(E)

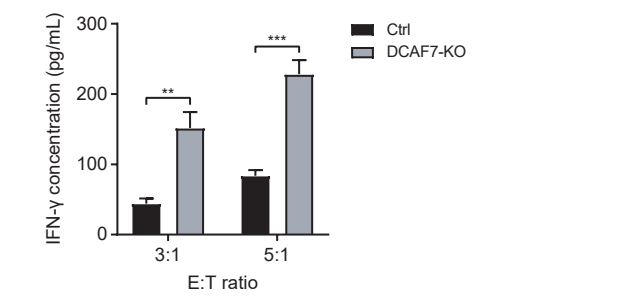

Figure S7

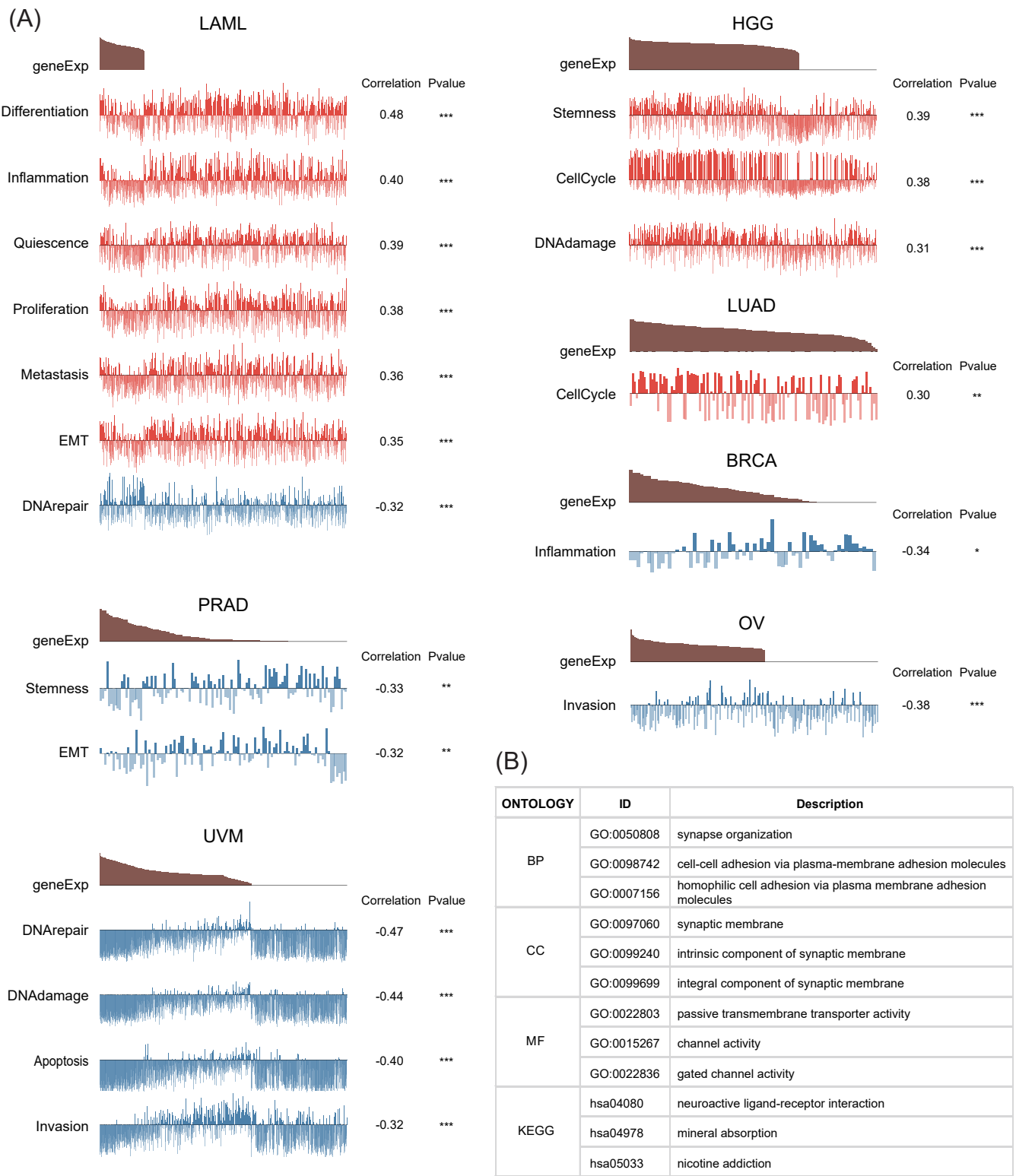

**Figure S8**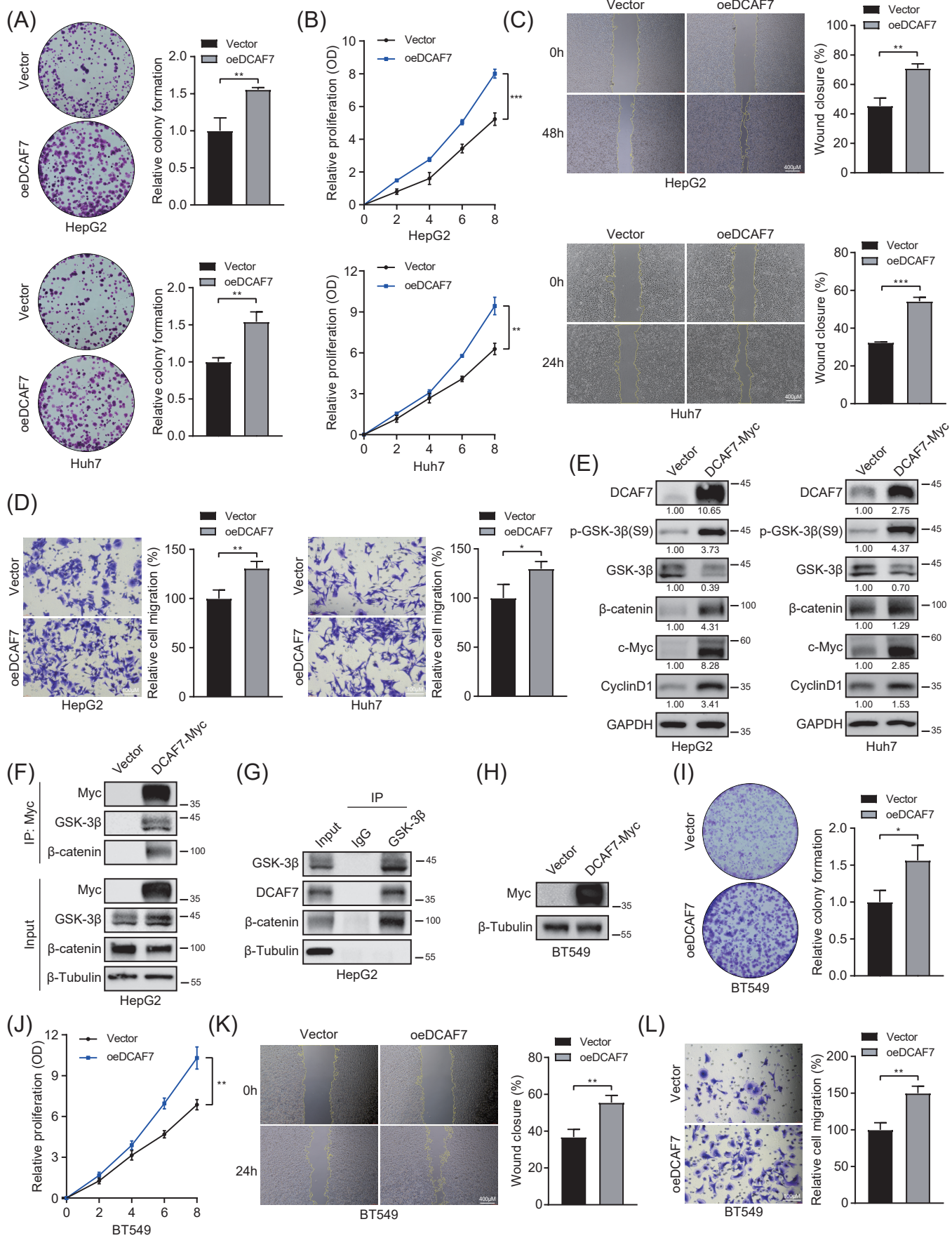

Supplement: Supplementary file 1 — Supporting information [file CTM2-16-e70572-s001.pdf]
